# Broad-spectrum kinome profiling identifies CDK6 upregulation as a driver of lenvatinib resistance in hepatocellular carcinoma

**DOI:** 10.1038/s41467-023-42360-w

**Published:** 2023-10-23

**Authors:** Carmen Oi Ning Leung, Yang Yang, Rainbow Wing Hei Leung, Karl Kam Hei So, Hai Jun Guo, Martina Mang Leng Lei, Gregory Kenneth Muliawan, Yuan Gao, Qian Qian Yu, Jing Ping Yun, Stephanie Ma, Qian Zhao, Terence Kin Wah Lee

**Affiliations:** 1 Department of Applied Biology and Chemical Technology, The Hong Kong Polytechnic University, Hong Kong, China; 2 School of Biomedical Sciences, The Chinese University of Hong Kong, Hong Kong, China; 3 State Key Laboratory of Cancer Biology, Biotechnology Center, School of Pharmacy, Fourth Military Medical University, Xi’An, China; 4 Department of Oncology, Tongji Hospital, Tongji Medical College, Huazhong University of Science and Technology, Wuhan, China; 5 Department of Pathology, Sun Yat-Sen University Cancer Center, Guangzhou, China; 6 School of Biomedical Sciences, Li Ka Shing Faculty of Medicine, The University of Hong Kong, Hong Kong, China; 7 State Key Laboratory of Liver Research, The University of Hong Kong, Hong Kong, China; 8 Research Institute for Future Food, The Hong Kong Polytechnic University, Hong Kong, China; 9 State Key Laboratory of Chemical Biology and Drug Discovery, The Hong Kong Polytechnic University, Hong Kong, China

**Keywords:** Cancer stem cells, Cancer therapy, Hepatology

## Abstract

Increasing evidence has demonstrated that drug resistance can be acquired in cancer cells by kinase rewiring, which is an obstacle for efficient cancer therapy. However, it is technically challenging to measure the expression of protein kinases on large scale due to their dynamic range in human proteome. We employ a lysine-targeted sulfonyl fluoride probe, named XO44, which binds to 133 endogenous kinases in intact lenvatinib-resistant hepatocellular carcinoma (HCC) cells. This analysis reveals cyclin-dependent kinase 6 (CDK6) upregulation, which is mediated by ERK/YAP1 signaling cascade. Functional analyses show that CDK6 is crucial in regulation of acquired lenvatinib resistance in HCC via augmentation of liver cancer stem cells with clinical significance. We identify a noncanonical pathway of CDK6 in which it binds and regulates the activity of GSK3β, leading to activation of Wnt/β-catenin signaling. Consistently, CDK6 inhibition by palbociclib or degradation by proteolysis targeting chimeras (PROTACs) is highly synergistic with lenvatinib in vitro. Interestingly, palbociclib not only exerts maximal growth suppressive effect with lenvatinib in lenvatinib-resistant HCC models but also reshapes the tumor immune microenvironment. Together, we unveil CDK6 as a druggable target in lenvatinib-resistant HCC and highlight the use of a chemical biology approach to understand nongenetic resistance mechanisms in cancer.

## Abstract (web summary)

Acquired resistance to cancer therapies can develop due to kinase rewiring. Here, using a broad-spectrum chemical probe, the authors perform kinome screening and identify CDK6-mediated accumulation of cancer stem cells as a driver of lenvatinib (multitargeted tyrosine kinase inhibitor) resistance in hepatocellular carcinoma.

## Introduction

Liver cancer (hepatocellular carcinoma, HCC) is one of the deadliest malignancies as it is the sixth most commonly diagnosed cancer and the fourth leading cause of cancer mortality in the world^1^. HCC treatment recently entered a new era with the development of molecular-targeted therapies, such as sorafenib, which improves the survival of advanced HCC patients. However, the survival benefit with sorafenib is modest with a median survival of 2.8 and 2.3 months longer than the placebo in two large-scale trials in Caucasians and Asians, respectively^2,3^. This unsatisfactory partial response is due to drug resistance^3,4^. Recently, lenvatinib, a multitargeted tyrosine kinase inhibitor (TKI) that selectively inhibits vascular endothelial growth factor receptor (VEGFR) 1–3, fibroblast growth factor receptor (FGFR) 1–4, platelet-derived growth factor receptor (PDGFR) *α*, RET, and KIT, has been approved by the FDA as a first-line treatment for unresectable advanced HCC patients. A recent phase III clinical trial of lenvatinib for HCC patients (NCT01761266) has demonstrated that lenvatinib is non-inferior to sorafenib in terms of overall survival in untreated advanced HCC^5^. Similar to other TKIs, the unsatisfactory survival benefits of lenvatinib may be due to acquired drug resistance. Therefore, studies on lenvatinib resistance mechanisms are urgently needed.

Increasing evidence has demonstrated that drug resistance can be acquired by cancer cells through kinase rewiring to replace the loss of signal in response to various TKIs. For instance, epidermal growth factor receptor (EGFR)-mutant lung cancer cells can acquire resistance to EGFR-TKIs, such as gefitinib, via HGF/MET-mediated activation of MAPK/extracellular signal-regulated kinase (ERK)1/2 and PI3K/AKT signaling^6^. Additionally, activation of insulin-like growth factor receptor (IGFR) 1β via upregulation of insulin-like growth factor II expression compensates for the loss of EGFR signaling impaired by gefitinib in colorectal cancer^7^. Therefore, it is of great importance to identify the kinases critical for this rewiring in response to lenvatinib treatment. However, it is technically challenging to measure the expression level of protein kinases on a large scale due to their dynamic range in the human proteome. Therefore, we applied a chemical proteomics approach using a lysine-targeted sulfonyl fluoride chemical probe, named XO44, which enables enrichment and accurate quantification of 133 endogenous kinases from live Jurkat cells^8^. For this purpose, we compared the kinase profiles between lenvatinib-resistant HCC cells and mock controls, which differ in self-renewing capacity, using this chemical probe. This analysis showed that cyclin-dependent kinase 6 (CDK6) was ranked at the top in both lenvatinib-resistant HCC cell lines compared to mock counterparts based on the average increase in fold-change. CDK6 belongs to the family of cyclin-dependent kinases, which is structurally and functionally similar to cyclin-dependent kinase 4 (CDK4), and its interactions with cyclin D1 (CCND1) play a crucial canonical role in cell cycle progression^9^. Intriguingly, CDK4, the binding partner of CDK6, showed no obvious alterations in lenvatinib-resistant cells, which revealed the distinct role of the noncanonical role of CDK6 in the regulation of lenvatinib resistance in HCC cells.

In this work, through using CRISPR activation and shRNA knockdown approaches, we find that CDK6 is critically involved in the regulation of cancer stemness and has clinical significance. During the process of acquired lenvatinib resistance, upregulation of CDK6 is mediated via the ERK/yes-associated protein (YAP) signaling cascade. Mechanistically, CDK6 noncanonically regulates lenvatinib resistance via direct binding and modulates the activity of GSK3β, leading to activation of the Wnt/β-catenin signaling pathway. CDK6 inhibition by palbociclib or CDK6 degradation by proteolysis targeting chimeras (PROTACs) synergizes with lenvatinib in vitro. Using in vivo lenvatinib-resistant HCC mouse models, palbociclib in combination with lenvatinib shows a maximal growth suppression effect. Interestingly, such combination treatment remodels immune landscape which favors immune suppression against HCC tumors. Collectively, the present study highlights the use of a chemical biology approach to uncover the adaptive resistance mechanisms in cancer.

## Results

### Quantitative kinome profiling identifies CDK6 upregulation in lenvatinib-resistant HCC cells

**Fig. 1:**
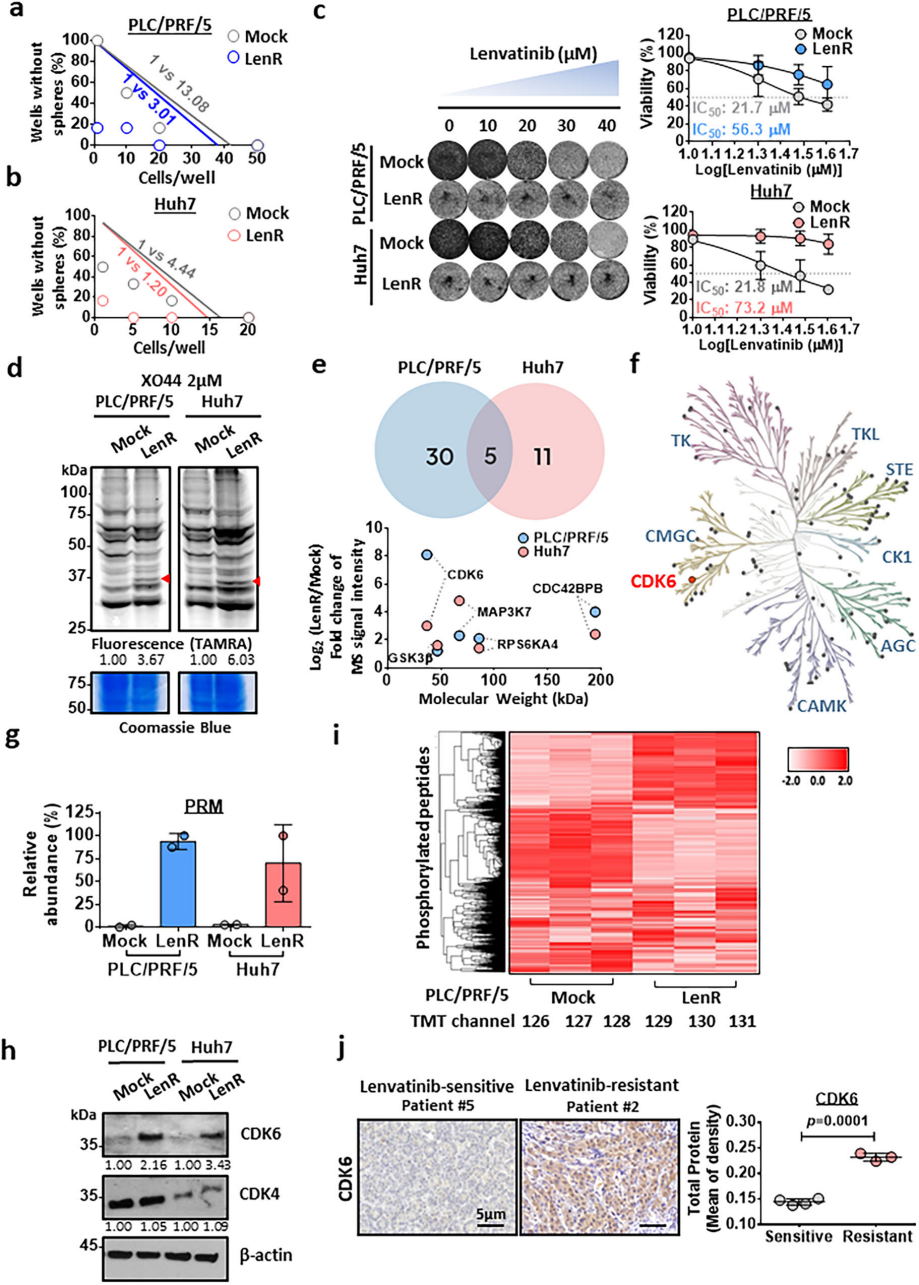
Broad-spectrum kinome profiling identification of CDK6 upregulation in lenvatinib-resistant HCC cells with enhanced self-renewal ability. **a**, **b** A limiting dilution assay showed that lenvatinib-resistant PLC/PRF/5 and Huh7 cells had enhanced self-renewal ability as evidenced by the increase in the stem cell frequency compared to the corresponding mock controls (LenR: lenvatinib-resistant) (*n* = 2 independent experiments, one-sided extreme limiting dilution analysis). **c** Long-term colony formation assay of mock and LenR PLC/PRF/5 and Huh7 cells. Cells were grown in the absence or presence of lenvatinib at the indicated concentrations, and they were then fixed and stained. The corresponding IC_50_ values were calculated and compared (*n* = 3 independent experiments). **d** The XO44 probe indicated upregulation of a kinase of approximately 36 kDa in lenvatinib-resistant PLC/PRF/5 and Huh7 cells compared to mock cells (PLC/PRF/5 *n* = 4 and Huh7 *n* = 3 independent experiments). **e** Venn diagram showing five upregulated proteins common to the two lenvatinib-resistant HCC cell lines with a 2-fold cutoff after mass spectrometry analysis (*n* = 1). CDK6 was found to be the most upregulated gene in lenvatinib-resistant PLC/PRF/5 while the second in Huh7. **f** Phylogenic kinase mapping illustrates the kinase profiles in LenR HCC cells. **g** Quantitation of CDK6 expression by the parallel reaction monitoring (PRM) approach (*n* = 2 independent experiments). **h** Western blot analysis showed that CDK6 protein was upregulated in lenvatinib-resistant PLC/PRF/5 and Huh7 cells. **i** Heatmap showing the changes in the phosphorylation levels of kinases in lenvatinib-resistant PLC/PRF/5 cell line (*n* = 3 independent experiments). **j** IHC staining showed the upregulation of CDK6 in lenvatinib-resistant HCC patients (*n* = 3 patients) when compared to sensitive group (*n* = 4 patients) (two-tailed *t* test). The protein expression was quantified using ImageJ. Data was presented as mean ± standard deviation. Source data are provided as a Source Data file.

We developed two lenvatinib-resistant HCC cell lines derived from the PLC/PRF/5 and Huh7 cell lines, which are endowed with self-renewal capacity (Fig. 1a, b). Lenvatinib-resistant clones of PLC/PRF/5 and Huh7 cells were established by continuous administration of gradually increasing lenvatinib concentrations up to 30 µM for up to 6 months. Successful establishment of lenvatinib resistance was evidenced by an observation of higher IC_50_ in lenvatinib-resistant cells compared to the corresponding mock counterparts upon lenvatinib treatment (Fig. 1c). We compared the difference in kinome profiles between these two cells using the broad-spectrum chemical probe named XO44 that specifically labels endogenous kinases. After treating live cells with XO44, the probe-labeled protein kinases were conjugated with rhodamine-azide via click chemistry, resolved by SDS-PAGE and then visualized by using a fluorescence scan (Fig. 1d). When most kinases remained unchanged, we observed a few protein bands with significantly enhanced fluorescence in lenvatinib-resistant HCC cell lines, and the protein bands had molecular weights between 30 and 37 kDa (indicated with arrows) (Fig. 1d). We next sought to confirm the identity of the altered protein kinase with a chemical proteomics approach utilizing the XO44 probe. The labeled protein kinases were conjugated to biotin-azide via a click reaction for affinity purification and subsequently analyzed with mass spectrometry. In total, 84 protein kinases from lenvatinib-resistant PLC/PRF/5 and Huh7 cells were quantified. Among the top five kinases with the highest fold increase in these cells, CDK6, which was ranked at the top, based on the average increase in fold-change in both cell lines (Fig. 1e, f, Supplementary Fig. 1). The molecular weight of CDK6 is 36.9 kDa, which was consistent with the protein band we observed in the in-gel fluorescence scan. To further verify the results acquired with shotgun proteomics, we employed an alternative mass spectrometry (MS) method, namely, parallel reaction monitoring (PRM), to quantify the change in CDK6 more accurately, and we observed a consistent increase in CDK6 in lenvatinib-resistant HCC (Fig. 1g). Western blot analysis confirmed this observation (Fig. 1h). Interestingly, CDK4 expression was not elevated in lenvatinib-resistant HCC cells and lenvatinib-resistant HCC xenograft tumor compared to the corresponding mock controls (Fig. 1h, Supplementary Fig. 2). Because CDK6 is an important protein kinase with various substrates, we evaluated the global phosphorylation change across the proteome with mass spectrometry. As expected, upregulated and downregulated phosphorylation levels were observed as downstream events of kinase alteration, indicating kinase reprogramming including CDK6 was a key event in generating lenvatinib resistance (Fig. 1i). To further examine whether the role of CDK6 in lenvatinib resistance is clinically relevant in HCC, we have examined CDK6 expression in tumors of seven HCC patients who received lenvatinib treatment. Among these patients, four of them were categorized as lenvatinib-sensitive while the remaining three were lenvatinib-resistant according to RECIST assessment (Supplementary Table 1). Upon analysis, we observed a significant upregulation of CDK6 in lenvatinib-resistant HCC patients when compared with sensitive group, further verifying the role of CDK6 in driving lenvatinib resistance in HCC (Fig. 1j).

### CDK6 regulates liver cancer stemness and is sporadically expressed in human HCCs with clinical significance

**Fig. 2:**
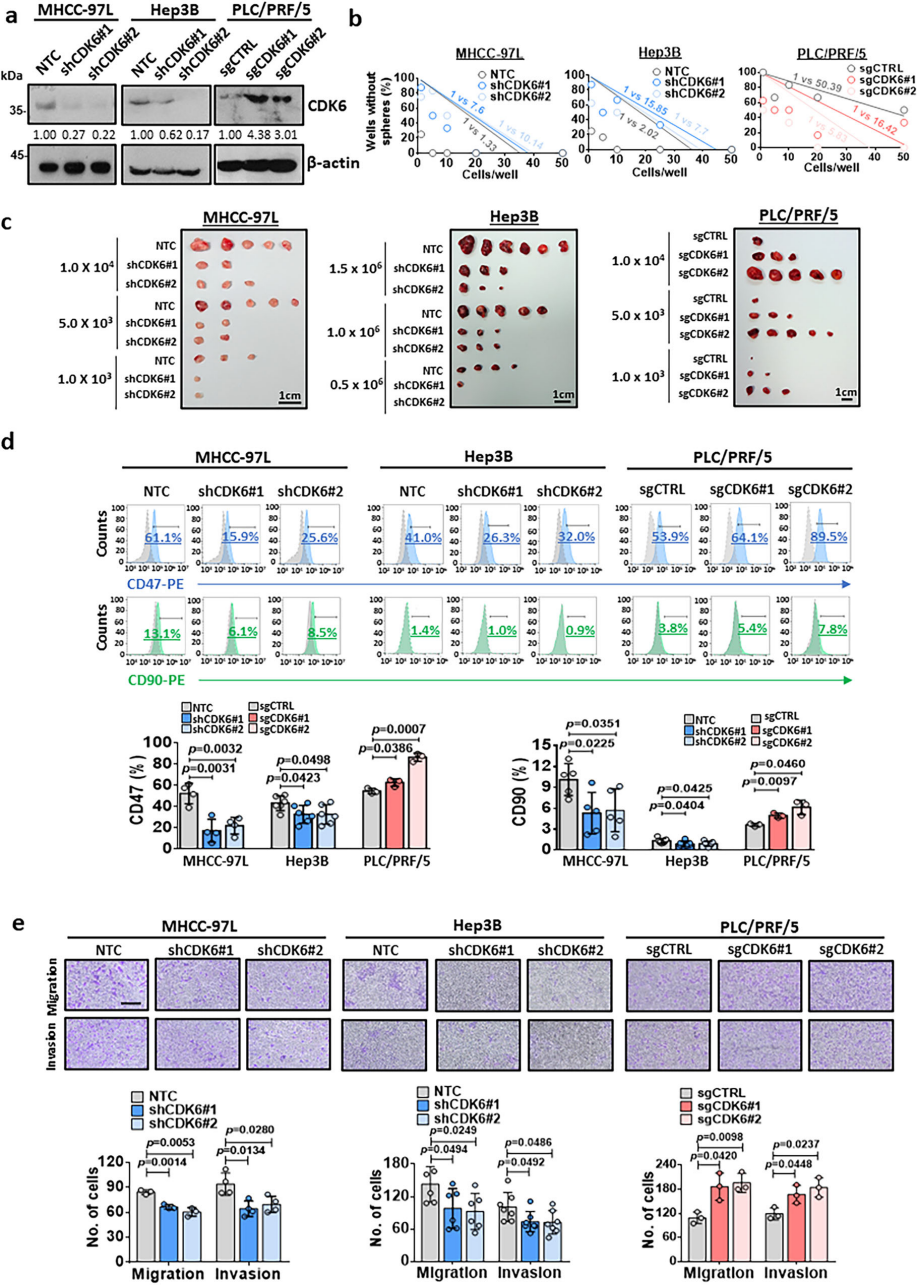
Critical role of CDK6 in the regulation of liver CSCs. **a** The CDK6 protein levels in the non-target control (NTC) and shCDK6 (#1 and #2) MHCC-97L and Hep3B cells as well as in the control (sgCTRL) and sgCDK6 (#1 and #2) subclones derived from PLC/PRF/5 cells were determined by western blot analysis (*n* = 3 independent experiments). **b** In vitro limiting dilution sphere analysis showed the role of CDK6 in the regulation of self-renewal ability (*n* = *2* independent experiments, one-sided extreme limiting dilution analysis). **c** Left and middle: Knockdown of CDK6 in MHCC-97L and Hep3B cell lines suppressed tumorigenicity compared to NTC cells. Right: Overexpression of CDK6 led to increased tumorigenicity of PLC/PRF/5 cells. Scale bar = 1 cm. **d** The expression of liver CSC markers, including CD47 (MHCC-97L: *n* = 4; Hep3B: *n* = 6 and PLC/PRF/5: *n* = 3 independent experiments) and CD90 (MHCC-97L: *n* = 5; Hep3B: *n* = 7 and PLC/PRF/5: *n* = 3 independent experiments), was measured by flow cytometry analysis (two-tailed *t* test)**. e** The migration and invasive abilities of HCC cells were evaluated by uncoated (top; MHCC-97L and PLC/PRF/5: *n* = 3 and Hep3B: *n* = 6 independent experiments) and Matrigel-coated transwell (bottom; MHCC-97L: *n* = 4; Hep3B: *n* = 7 and PLC/PRF/5: *n* = 3 independent experiments) assays, respectively. Scale bar = 250 µm. (two-tailed *t* test). Data was presented as mean ± standard deviation. Source data are provided as a Source Data file.

**Fig. 3:**
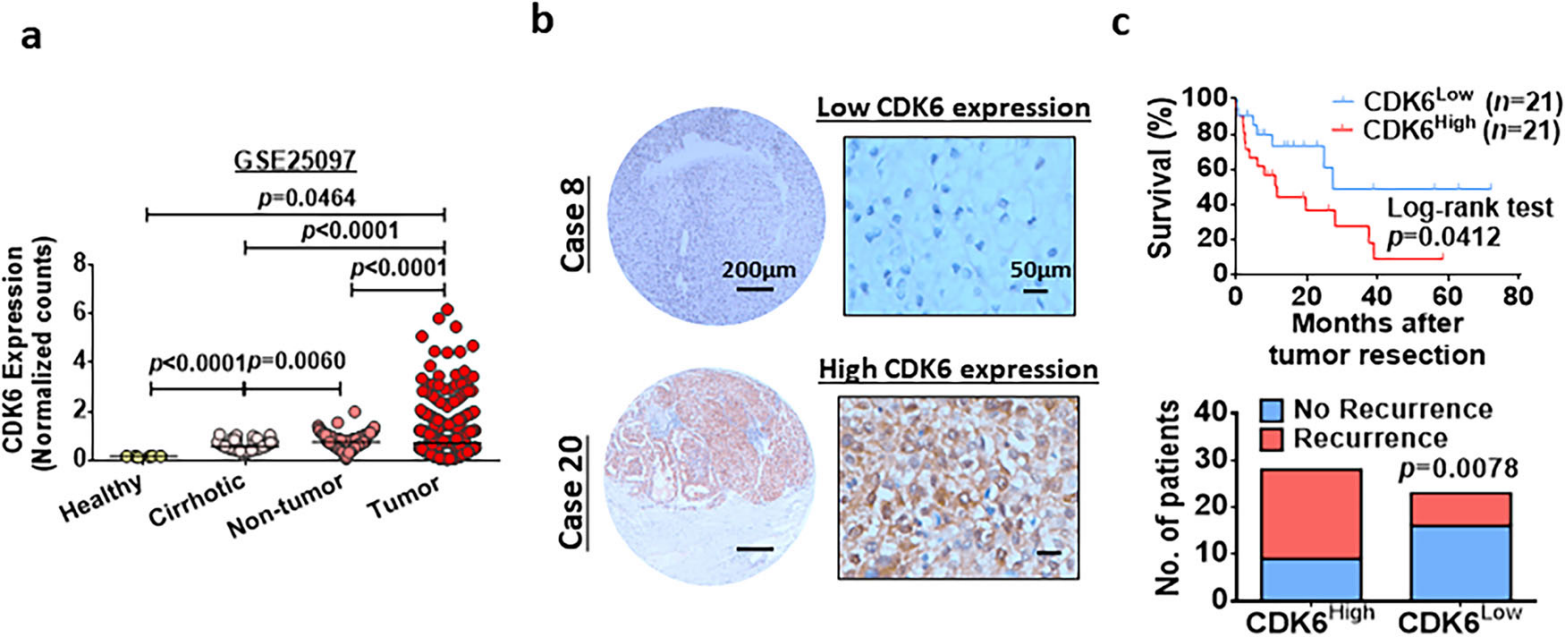
Clinical relevance of CDK6 in HCC. **a** By analysis of the GSE25097 dataset^10^, we found upregulation of *CDK6* mRNA in a cohort of human HCC samples (tumor *n* = 268 and adjacent non-tumor *n* = 243). In addition, *CDK6* was upregulated in cirrhotic samples (*n* = 40) compared to healthy donors (*n* = 6) (two-tailed *t* test). **b** A tissue microarray consisting of 51 tumor tissues and corresponding non-tumor liver tissues was subjected to IHC analysis. Case 8 showed low expression of CDK6, while case 20 showed high expression of this protein. Scale bars = 50 µm and 200 µm. **c** Patients with high CDK6 expression (*n* = 21) had shorter disease-free survival than those with low expression (*n* = 21) *(p* = 0.0412; log-rank test). High-CDK6 expression was significantly correlated with HCC recurrence (*p* = 0.0078; *χ*^2^ test). Data was presented as mean ± standard deviation. Source data are provided as a Source Data file.

CDK6 was upregulated in self-renewing lenvatinib-resistant HCC cells (Fig. 1), indicating its role in the regulation of cancer stemness in HCC. To examine whether CDK6 functionally drives self-renewal and tumor formation, we manipulated CDK6 expression in HCC cells using lentiviral-based knockdown and the CRISPR/dCas9-VP64-p65-Rta gene activation system (Dharmacon). Western blot analysis demonstrated high expression of CDK6 in a panel of HCC cell lines, including MHCC-97L and Hep3B cells (Supplementary Fig. 3). Therefore, these two cell lines were selected for the knockdown experiment, while PLC/PRF/5 cells were selected for the overexpression experiment. Western blot analysis confirmed the successful establishment of the knockdown and overexpression clones (Fig. 2a). Using a limiting dilution assay, CDK6 knockdown reduced the stem cell frequencies of MHCC-97L and Hep3B cells. In contrast, CDK6 overexpression enhanced the stem cell frequency of PLC/PRF/5 cells (Fig. 2b). In an in vivo tumorigenicity assay, knockdown of CDK6 significantly reduced the size and number of tumors formed (Fig. 2c, Supplementary Table 2). Consistent with our findings, CDK6 overexpression in PLC/PRF/5 cells significantly enhanced tumorigenicity with an increase in the estimated cancer stem cell (CSC) frequency (Fig. 2c, Supplementary Table 2). Consistently, alterations in CDK6 changed the expression levels of four known liver CSC markers namely, CD47, CD90, CD133, and EpCAM (Fig. 2d, Supplementary Fig. 4a), and pluripotency-related markers including SOX2 and OCT4 (Supplementary Fig. 4b). In addition, CDK6 was found to play a regulatory role in the migration and invasion abilities of HCC cells (Fig. 2e). By analyzing the GSE25097 dataset^10^, a publicly available dataset, we found a stepwise increase in CDK6 expression from normal to cirrhosis to progressive HCC stages, suggesting an oncogenic role for CDK6 in liver carcinogenesis (Fig. 3a). To further confirm CDK6 overexpression in HCC, we used a tissue microarray consisting of 51 HCC samples and the corresponding matched non-tumor liver tissue samples to evaluate CDK6 expression by immunohistochemical staining (Fig. 3b, Supplementary Table 3). Patients with high CDK6 expression had shorter disease-free survival (*p* = 0.0412, respectively; log-rank test) and a higher recurrence rate (*p* = 0.0078) (Fig. 3c).

### Genetic suppression and pharmacological inhibition of CDK6 sensitize the effect of lenvatinib in HCC cells

**Fig. 4:**
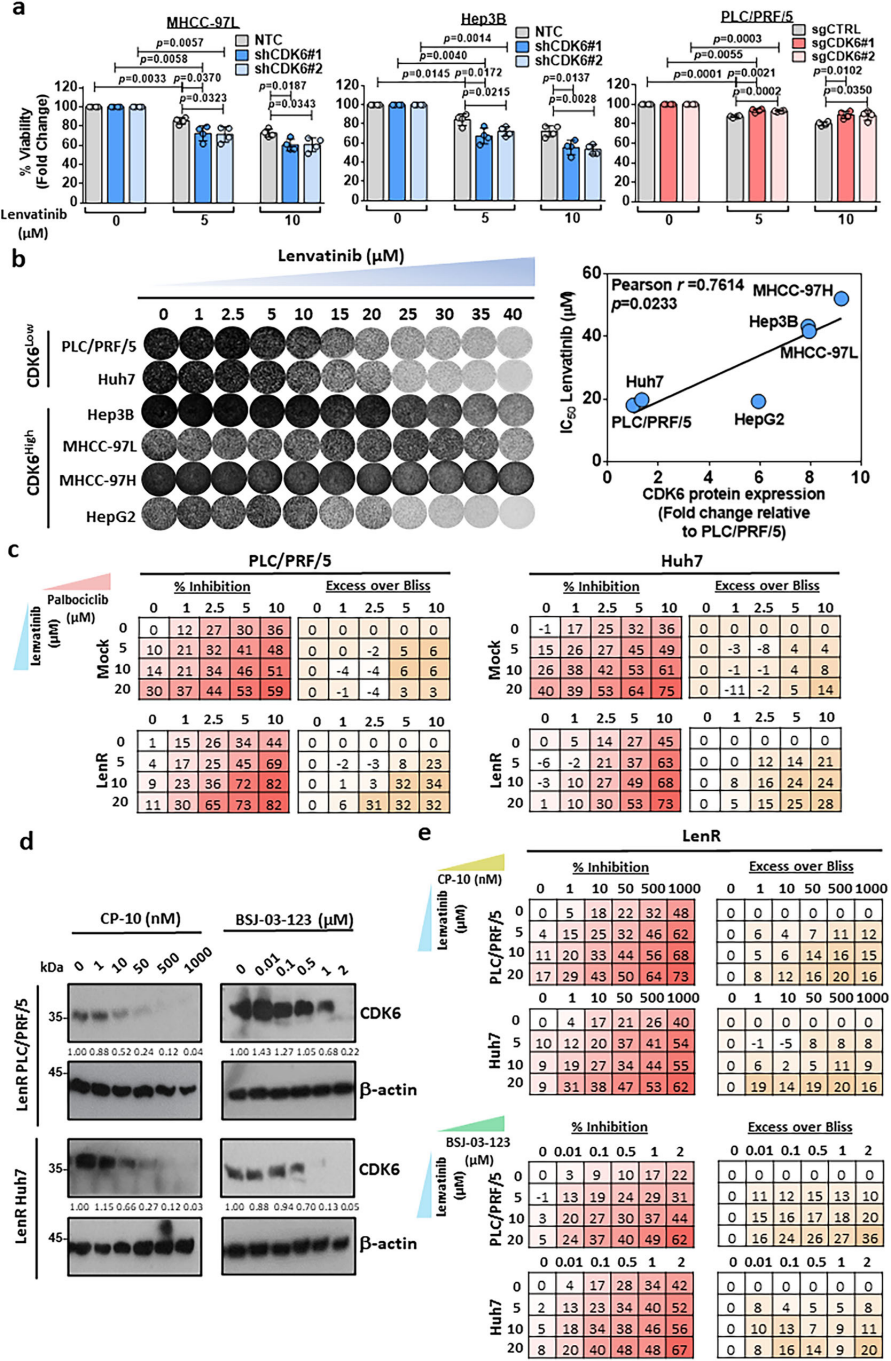
CDK6 is a critical determinant in lenvatinib resistance. **a** The cell viability of shCDK6 (MHCC-97L and Hep3B) and sgCDK6 (PLC/PRF/5) under treatment of lenvatinib (5 µM and 10 µM) for 48 hours was evaluated by MTT assay (*n* = 4 independent experiments, two-tailed *t* test). **b** Long-term colony formation assay of six HCC cell lines. Cells were grown in the absence or presence of lenvatinib at the indicated concentrations for 10–14 days, fixed, and stained. A positive correlation between the IC_50_ values of lenvatinib and CDK6 protein levels was observed in six HCC cell lines (Pearson *r* = 0.7614, *p* = 0.0233). **c** Combined treatment of lenvatinib and palbociclib synergistically inhibited the growth of mock and lenvatinib-resistant PLC/PRF/5 and Huh7. Cells were treated with the indicated combination at different doses for 48 hours. Cell viability was measured by MTT assay (*n* = 3 independent experiments). Positive value in excess over Bliss indicated a synergistic effect in the combined treatment. **d** CP-10 at 50 nM effectively degraded the expression of CDK6 in lenvatinib-resistant PLC/PRF/5 and Huh7 cells, while 2 µM BSJ-03-123 was sufficient to degrade CDK6 in these cells. The protein expression was quantified using ImageJ, normalized to β-actin expression, and expressed as fold-change relative to no treatment control. Representative images of *n* = 3 independent experiments. **e** Lenvatinib combined with either CP-10 for 5 days or BSJ-03-123 for 6 days synergistically suppressed the growth of lenvatinib-resistant PLC/PRF/5 and Huh7 cells. Cell viability was measured by MTT assay (*n* = 3 independent experiments). Positive value in excess over Bliss indicated a synergistic effect in the combined treatment. Data was presented as mean ± standard deviation. Source data are provided as a Source Data file.

Given that CDK6 upregulation was found in lenvatinib-resistant HCC cells, we investigated whether CDK6 suppression resulted in the sensitization of HCC cells to lenvatinib treatment. MTT assay demonstrated decreased survival and reduced resistance to lenvatinib following CDK6 knockdown. In contrast, CDK6 overexpression conferred drug resistance in PLC/PRF/5 cells (Fig. 4a). In a panel of HCC cells with variable degrees of CDK6 expression, there was a positive correlation between CDK6 levels and lenvatinib resistance (Fig. 4b). The CSC- and survival-enhancing effects of CDK6 were kinase-dependent as transfection of the kinase-dead mutant, CDK6-K43M, did not augment the self-renewal ability nor the rescue apoptotic effect of lenvatinib treatment respectively (Supplementary Fig. 5a–d). Palbociclib, a CDK4/6 inhibitor, showed a synergistic effect when combined with lenvatinib (Fig. 4c). To further confirm that the sensitization effect of palbociclib is not dependent on CDK4, we examined the combination effect of PROTAC against CDK6 (CP-10^11^ and BSJ-03-123^12^) with lenvatinib in lenvatinib-resistant PLC/PRF/5 and Huh7 cells and high CDK6-expressing MHCC-97L and Hep3B cells. Western blot showed degradation of CDK6 in these cells in dose-dependent manner (Fig. 4d, Supplementary Fig. 6a). Furthermore, the MTT assay demonstrated that either CP-10 or BSJ-03-123 combined with lenvatinib synergistically suppressed the growth of lenvatinib-resistant PLC/PRF/5 and Huh7 cells and high CDK6-expressing HCC cells (Fig. 4e, Supplementary Fig. 6b).

### CDK6 upregulation is mediated by ERK/YAP signaling in lenvatinib-resistant HCC cells

**Fig. 5:**
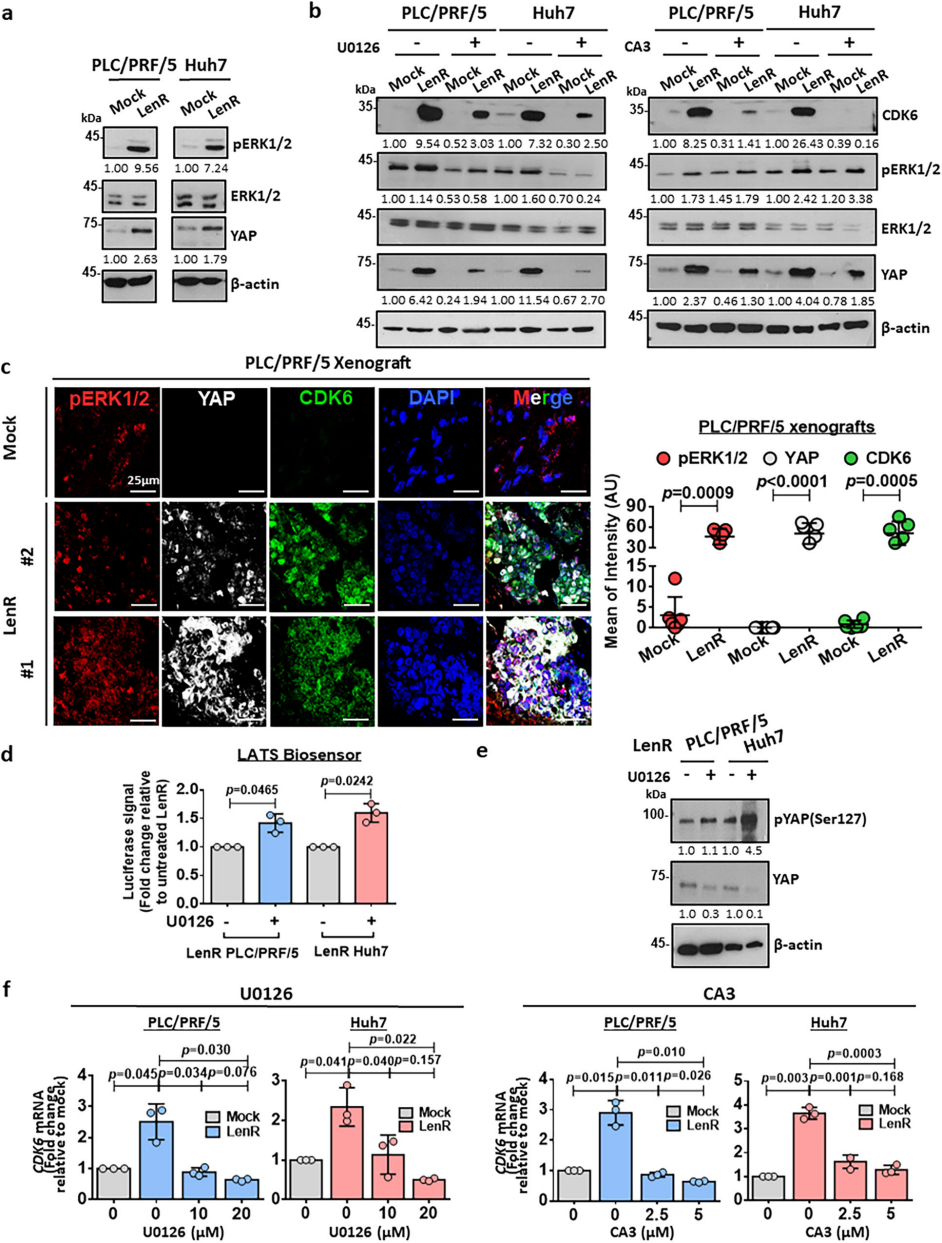
CDK6 is downstream of the ERK/YAP signaling pathway in HCC. **a** pERK1/2 and YAP were upregulated in both lenvatinib-resistant PLC/PRF/5 and Huh7 cells. Representative images of *n* = 3 independent experiments. **b** A MEK inhibitor (U0126) suppressed pERK1/2 levels, which was accompanied by decreased expression of YAP and CDK6 in mock and lenvatinib-resistant PLC/PRF/5 and Huh7 cells. A YAP inhibitor (CA3) suppressed CDK6 expression in these cells. Representative images of *n* = 3 independent experiments. **c** Lenvatinib-resistant PLC/PRF/5-derived tumors showed a significant upregulation of pERK1/2, YAP, and CDK6 expression when compared with mock tumors. A positive Pearson’s correlation coefficient (pERK1/2 & YAP: *r* = 0.212; pERK1/2 & CDK6: *r* = 0.262; YAP & CDK6: *r* = 0.394) showed the colocalization among these three proteins in lenvatinib-resistant PLC/PRF/5-derived tumors. pERK1/2 staining (red), YAP staining (white), CDK6 staining (green), and DAPI staining (blue). Quantification of fluorescent intensity (*n* = 5 random fields, two-tailed *t* test) and Pearson’s correlation coefficient (*n* = 6 random fields with mean of the coefficient being indicated) was performed using ImageJ software. Scale bar = 25 μm. **d** LATS-Biosensor luciferase assay showed an increase in LATS activity in lenvatinib-resistant cells upon U0126 treatment (*n* = 3 independent experiments, two-tailed *t* test). **e** Western blot analysis showed an upregulation of phosphorylated YAP at Ser127 while total YAP level was suppressed, when pERK1/2 was abated by U0126 in lenvatinib-resistant cells. **f** U0126 at 10 µM and 20 µM and CA3 at 2.5 µM and 5 µM suppressed the mRNA expression of *CDK6* (*n* = 3 independent experiments, two-tailed *t* test). Data was presented as mean ± standard deviation. Source data are provided as a Source Data file.

CDK6 has been previously reported to be upregulated by YAP1^13^, while YAP expression is regulated by ERK1/2^14^. Along with CDK6 upregulation in lenvatinib-resistant HCC cells, the expression of pERK1/2 and YAP was also elevated (Fig. 5a). Likewise, both YAP and pERK1/2 were overexpressed in high CDK6-expressing HCC cell lines when compared with low CDK6 counterparts (Supplementary Fig. 7). To further confirm that ERK/YAP signaling is the upstream regulator of CDK6 expression, we treated lenvatinib-resistant PLC/PRF/5 and Huh7 cells with MEK inhibitor (U0126). Consistently, the expression of CDK6 and YAP was decreased to levels similar to those of the mock control upon treatment with these inhibitors (Fig. 5b, Supplementary Fig. 8a). A similar decrease in CDK6 expression was observed in the same HCC cells treated with the YAP inhibitor (CA3) (Fig. 5b, Supplementary Fig. 8b). The colocalization of these three proteins in both cytoplasmic and nuclear compartments was further confirmed in lenvatinib-resistant PLC/PRF/5 xenografts using confocal immunofluorescence (IF) microscopy (Fig. 5c). To further explore how ERK1/2 activates YAP to drive the upregulation of CDK6, we examined the activity of one of the upstream negative regulators of YAP protein, LATS1/2, using LATS-Biosensor assay^15^. LATS-Biosensor monitors the activity of LATS1/2 through the interaction between the phosphorylated YAP at Ser127 (pYAPSer127) by LATS1/2 and 14-3-3 protein. 14-3-3 protein binds specifically to phosphorylated, but not unphosphorylated Ser127-YAP^16^. The transactivation of downstream gene targets of YAP is suppressed after the pYAP (Ser127) binds to 14-3-3 protein. Upon ERK1/2 inhibition using U0126, the LATS1/2 activity was found to be increased (Fig. 5d). This observation, together with upregulation of pYAP (Ser127) protein level (Fig. 5e), further confirms the regulatory role of ERK1/2 on YAP via suppression of LATS1/2. Next, we examined whether CDK6 is transcriptionally upregulated in lenvatinib-resistant HCC cells. Quantitative reverse transcription polymerase chain reaction (qRT-PCR) analysis demonstrated that *CDK6* mRNA was upregulated in lenvatinib-resistant HCC cells, while its level was decreased upon treatment with U0126 and CA3 (Fig. 5f). In clinical HCC samples, a similar correlation was observed among *YAP1* and *ERK2* with *CDK6* (Supplementary Fig. 9).

### CDK6 directly binds and phosphorylates GSK3β, leading to Wnt/β-catenin activation

**Fig. 6:**
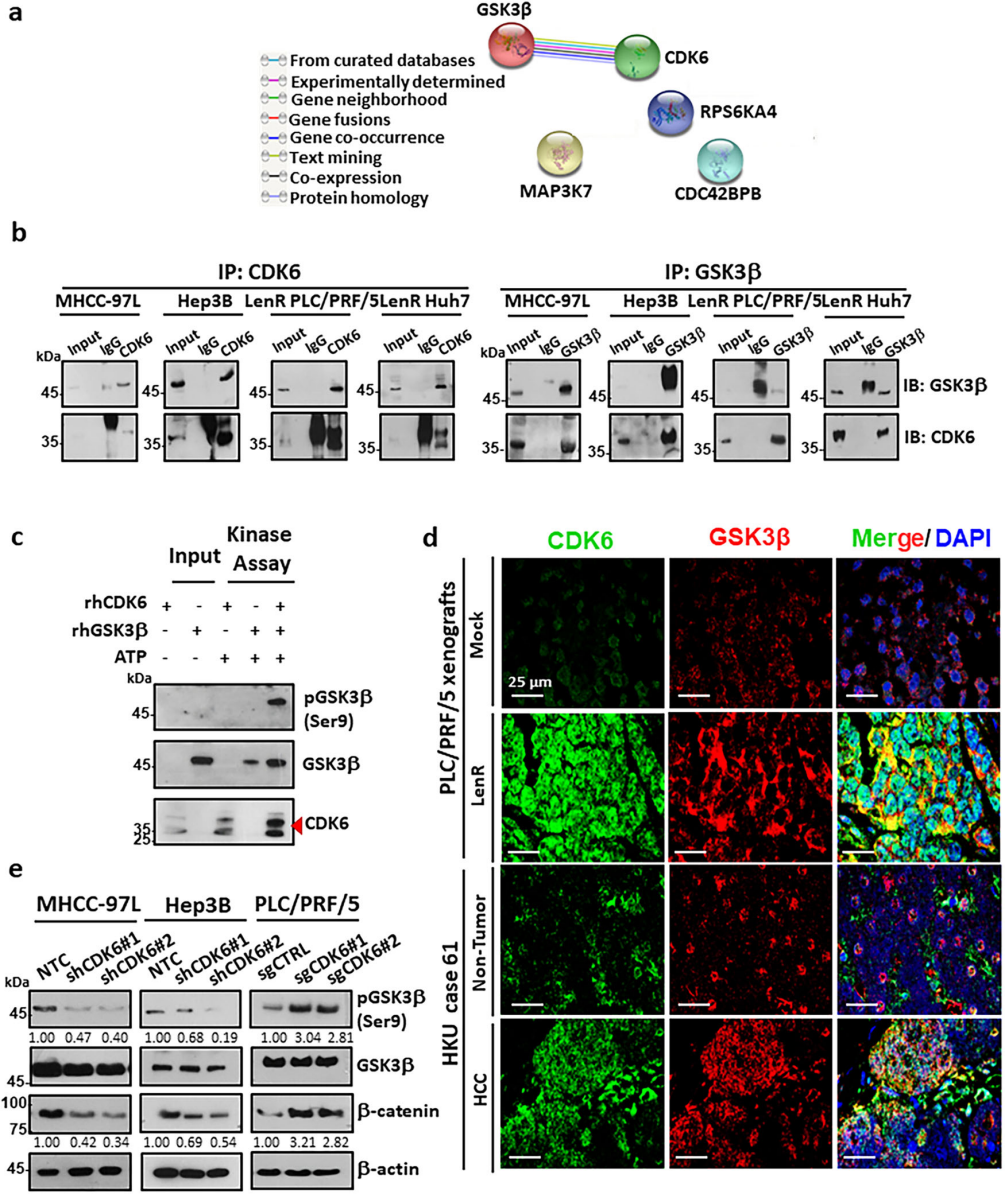
Physiological significance of the CDK6-GSK3β interaction. **a** Protein-protein interactions of enhanced kinases common to lenvatinib-resistant PLC/PRF/5 and Huh7 cells was generated using the STRING database. Lines are colored according to the type of association between the indicated proteins (see legend). **b** Reciprocal coimmunoprecipitation demonstrated the interaction between endogenous CDK6 and GSK3β in high CDK6-expressing MHCC-97L and Hep3B cells and lenvatinib-resistant cells (*n* = 2 independent experiments). **c** Kinase assay using recombinant human CDK6 (rhCDK6) and GSK3β (rhGSK3β) confirmed that GSK3β as a direct substrate of CDK6, as evidenced by the phosphorylation of GSK3β at serine 9 (*n* = 2 independent experiments). **d** In a lenvatinib-resistant PLC/PRF/5-derived tumor (*n* = 1) and HCC clinical tumor specimen (case 61, *n* = 1), colocalization between CDK6 and GSK3β was observed in HCC cells. CDK6 staining (green), GSK3β staining (red), and DAPI staining (blue). Scale bar = 25 μm. **e** Western blot analysis of GSK3β, p-GSK3β(Ser9) and β-catenin was performed in CDK6-knockdown and CDK6-overexpressing HCC cells. β-actin was used as a normalization control (*n* = 3 independent experiments). Source data are provided as a Source Data file.

**Fig. 7:**
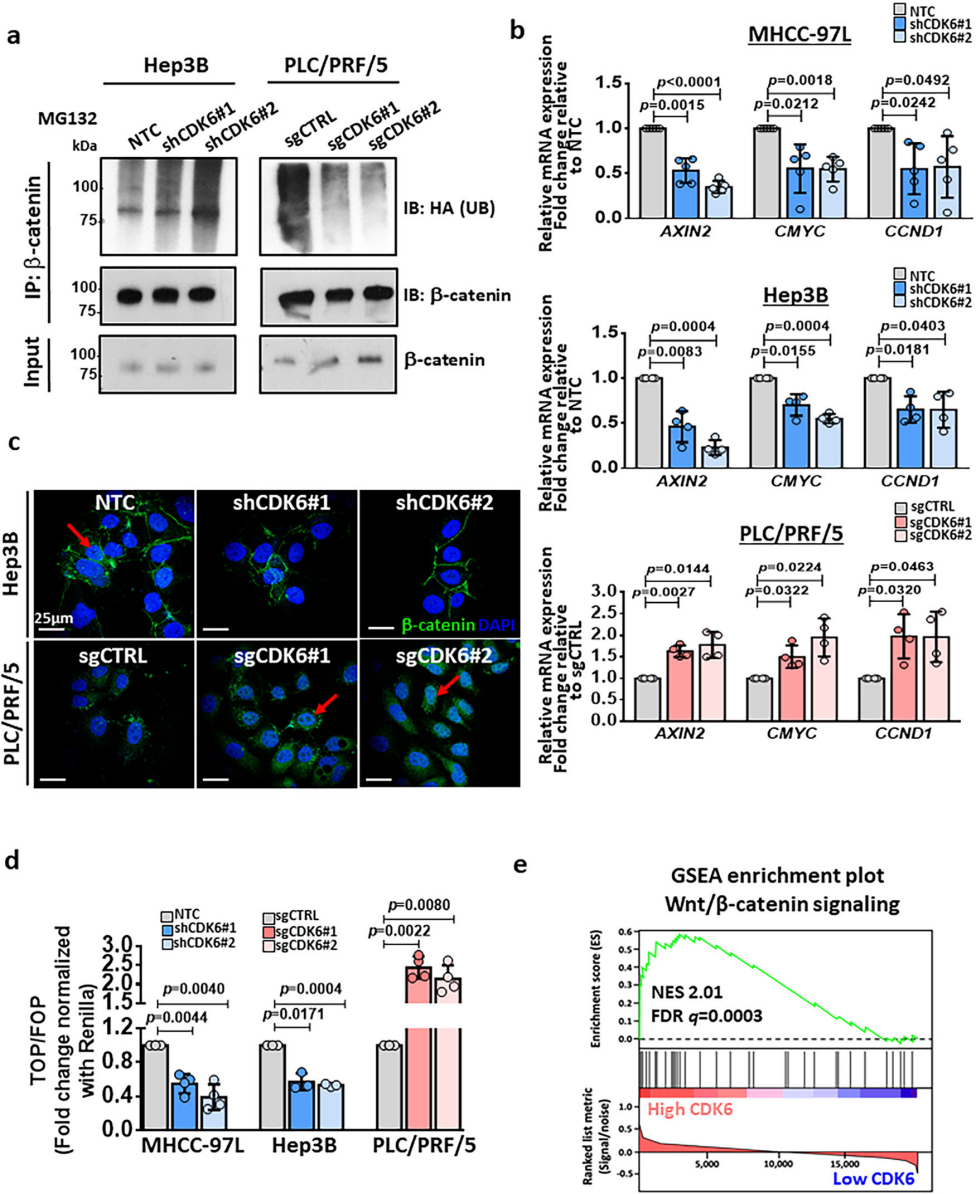
Functional role of CDK6 in activation of Wnt/β-catenin signaling cascade. **a** After treatment with 10 μM MG132 for 8 hours, β-catenin was pulled down to investigate the effect of CDK6 on the abundance of ubiquitinated β-catenin. Knockdown of CDK6 promoted the ubiquitination of β-catenin, while overexpression significantly suppressed ubiquitination (IP: β-catenin, IB: HA; *n* = 3 independent experiments). **b** The expression of β-catenin downstream genes, including *AXIN2*, *CMYC* and *CCND2*, was examined in CDK6-silencing and overexpressing HCC cells (MHCC-97L: *n* = 5; Hep3B and PLC/PRF/5: *n* = 4 independent experiments, two-tailed *t* test). **c** The effect of CDK6 alteration on β-catenin accumulation (*n* = 2 independent experiments). Arrows indicate nuclear expression of β-catenin. β-catenin staining (green) and DAPI staining (blue). Scale bar = 25 μm. **d** Using the β-catenin TCF binding TOP/FOPFLASH luciferase reporter assay, the transactivating activity of β-catenin was examined in CDK6-knockdown and overexpressing HCC cells (MHCC-97L and PLC/PRF/5: *n* = 4 and Hep3B: *n* = 3 independent experiments, two-tailed *t* test). **e** By TCGA data analysis, we found that Wnt/β-catenin was enriched in HCC patients with high CDK6 expression with a normalized ES score of 2.011 (FDR *q* value of 0.0003). Data was presented as mean ± standard deviation. Source data are provided as a Source Data file.

STRING analysis of the kinome profiles in lenvatinib-resistant HCC cells (Fig. 1e) showed that CDK6 had a potential protein-protein interaction with GSK3β (Fig. 6a). Based on this finding, we examined the interaction between CDK6 and GSK3β by reciprocal immunoprecipitation using endogenous proteins in high-CDK6-expressing MHCC-97L and Hep3B cells and lenvatinib-resistant HCC cells. Immunoprecipitation assays revealed a physical interaction between CDK6 and GSK3β in these cells (Fig. 6b). We further confirmed that GSK3β as a direct substrate for CDK6 evidenced by phosphorylation of GSK3β at Ser9 through in vitro kinase assay (Fig. 6c). The colocalization of these two proteins was further confirmed in lenvatinib-resistant PLC/PRF/5 xenografts and one pair of HCC clinical samples using confocal IF microscopy (Fig. 6d). We next examined whether CDK6 regulates the phosphorylation of GSK3β at serine 9 via direct binding. Western blot analysis demonstrated that suppression of CDK6 inhibited phospho-GSK3β (Ser9) and β-catenin expression, while the opposite effects were observed after CDK6 overexpression (Fig. 6e). The effect of CDK6 on GSK3β/β-catenin signaling may be independent to p53 status, as evidenced by the similar suppression of phosphorylation of GSK3β at Ser9 and β-catenin in shCDK6 HepG2 cells with wild-type p53 expression (Supplementary Fig. 10a). Given the role of GSK3β in β-catenin degradation by ubiquitination, we showed that CDK6 knockdown in HCC cells increased β-catenin ubiquitination, as determined by ubiquitination and western blot assays (Fig. 7a), reduced transcription of Wnt target genes (including *AXIN2*, *CMYC* and *CCND2*) (Fig. 7b), reduced β-catenin accumulation and decreased transactivating activity of β-catenin as determined by a TOP/FOP reporter assay (Fig. 7c, d). Opposite effects on β-catenin expression, ubiquitination and transactivation activity were observed in CDK6-overexpressing cells (Fig. 7a–d). We further examined whether β-catenin is the downstream effector of CDK6-mediated lenvatinib resistance by suppressing β-catenin expression in lenvatinib-resistant PLC/PRF/5 and Huh7 cells. Annexin-V staining demonstrated decreased survival and reduced lenvatinib resistance following β-catenin knockdown (Supplementary Fig. 10b). Gene set enrichment analysis (GSEA) of The Cancer Genome Atlas (TCGA) demonstrated that the Wnt/β-catenin signaling pathway was significantly enriched in HCC patients with high *CDK6* expression, which further confirmed Wnt/β-catenin as the major effector of CDK6 signaling in the in vitro findings (Fig. 7e). The above results suggested that CDK6 regulates the Wnt/β-catenin signaling pathway by directly regulating the activity of GSK3β.

### Palbociclib combined with lenvatinib results in maximal tumor growth suppression in a lenvatinib-resistant HCC xenograft model

**Fig. 8:**
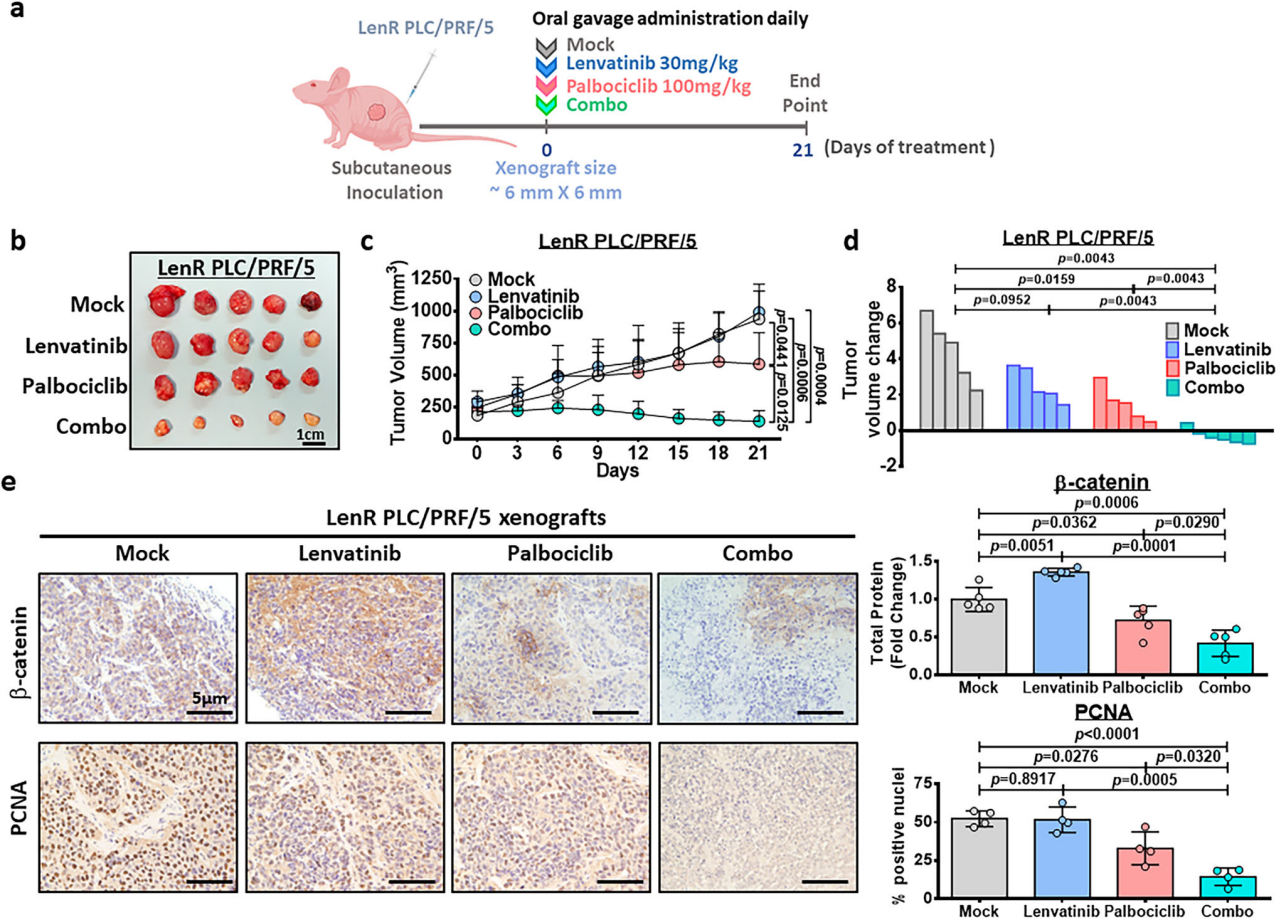
The effect of palbociclib/lenvatinib treatment in suppressing tumor growth in LenR HCC xenografts. **a** Schematic diagram of the treatment regimen of mock (0.5% methylcellulose in saline), palbociclib (100 mg/kg), lenvatinib (30 mg/kg) and the combined treatment (combo) group. **b** Response of lenvatinib-resistant PLC/PRF/5 xenografts to treatment with lenvatinib (30 mg/kg daily), palbociclib (100 mg/kg daily), or their combination through oral administration. Harvested tumors are shown. Scale bar = 1 cm. Five representative tumors in each group were shown. **c** Tumor growth curve showing the response towards each treatment (*n* = 5 mice in mock, lenvatinib and palbociclib while *n* = 6 mice for combo; two-tailed *t* test). **d** Waterfall plot showing the response of each tumor after 21 days (two-tailed Mann–Whitney *U* test). **e** Immunohistochemical images of β-catenin (*n* = 5) and PCNA (*n* = 4) in resected tumors (two-tailed *t* test). Scale bar = 5 µm. Protein signal intensity and number of positive nuclei was quantified using ImageJ software. Data was presented as mean ± standard deviation. Source data are provided as a Source Data file.

We examined the therapeutic effect of palbociclib alone and its combined effect with lenvatinib in vivo using HCC xenografts derived from lenvatinib-resistant PLC/PRF/5 cells. Treatment was started once the size of the xenograft reached ~6 mm × 6 mm (length × width). The mice were separated into the following four subgroups: (i) mock (0.5% methylcellulose in saline); (ii) lenvatinib (30 mg/kg) in water; (iii) palbociclib (100 mg/kg) in 0.5% methylcellulose in saline; and (iv) palbociclib and lenvatinib (Fig. 8a). The tumors and their corresponding volumes after treatment for 21 days are shown in Fig. 8b, c. Successful establishment of lenvatinib-resistant PLC/PRF/5 cells was confirmed by the observation that lenvatinib failed to suppress tumor growth, accompanied with high CDK6 expression (Supplementary Fig. 11). Moreover, palbociclib suppressed tumor growth to a certain extent. Palbociclib combined with lenvatinib exerted a synergistic effect, resulting in maximal suppression of tumor growth compared to that of the control group. Importantly, we found that this combination treatment significantly reduced the tumor volumes by 34.9%, relative to the original tumor volume on day 0 (Fig. 8d). During this experiment, no signs of toxicity (infection, diarrhea or loss of body weight) were observed in the animals undergoing combination treatment (Supplementary Fig. 12). Consistent with these biological effects, total β-catenin was reduced in the combination treatment group (Fig. 8e). In addition, the combination treatment significantly suppressed cell proliferation as evidenced by a decrease of positive nuclei in proliferating cell nuclear antigen (PCNA) staining compared to single-agent treatment and mock control (Fig. 8e).

### Palbociclib sensitizes HCC cells to lenvatinib treatment in a lenvatinib-resistant *Trp53*^KO^/*MYC*^OE^ HCC mouse model

**Fig. 9:**
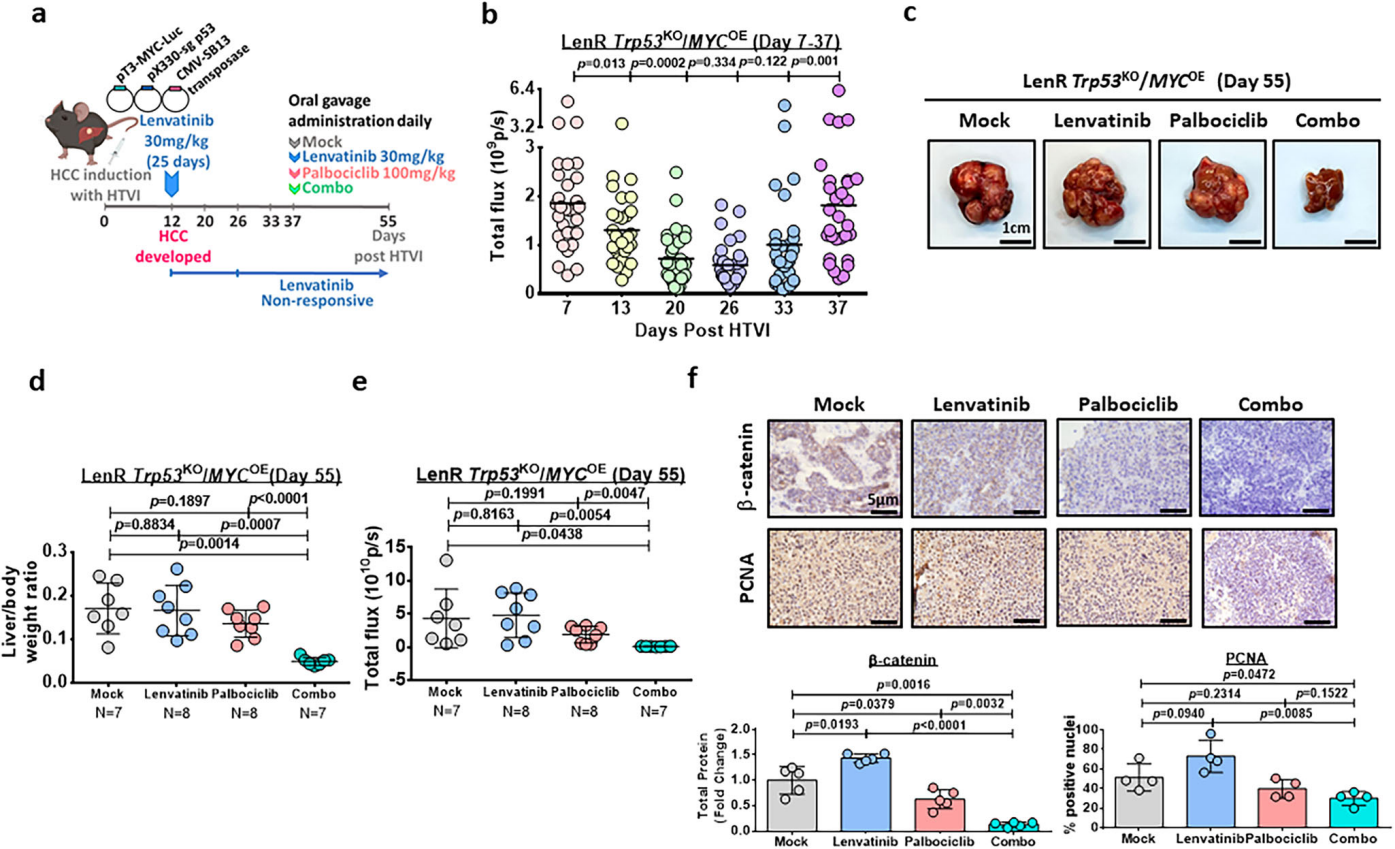
The effect of palbociclib/lenvatinib treatment in suppressing tumor growth in an immune-competent mouse model. **a** Schematic diagram of the treatment regimen of mock (0.5% methylcellulose in saline), palbociclib (100 mg/kg), lenvatinib (30 mg/kg) and the combined treatment (combo). **b** Luciferase intensity indicates the successful establishment of lenvatinib-resistant HCC tumors upon administration of lenvatinib for 25 days (*n* = 30 mice, two-tailed Mann–Whitney *U* test). **c** Representative images of HCC tumors derived from the four groups at the endpoint. Scale bar = 1 cm. **d** Graph showing the liver/body weight ratio generated from mice at the endpoint (*n* = 7 mice for mock and combo while *n* = 8 mice for lenvatinib and palbociclib, two-tailed *t* test). **e** The signal intensity of the livers in the four groups is shown (*n* = 7 mice for mock and combo while *n* = 8 mice for lenvatinib and palbociclib, two-tailed *t* test). **f** Immunohistochemical images of β-catenin (*n* = 5) and PCNA (*n* = 4) in resected tumors (two-tailed *t* test). Scale bar = 5 µm. Protein signal intensity and number of positive nuclei were quantified using ImageJ. Data was presented as mean ± standard deviation. Source data are provided as a Source Data file.

**Fig. 10:**
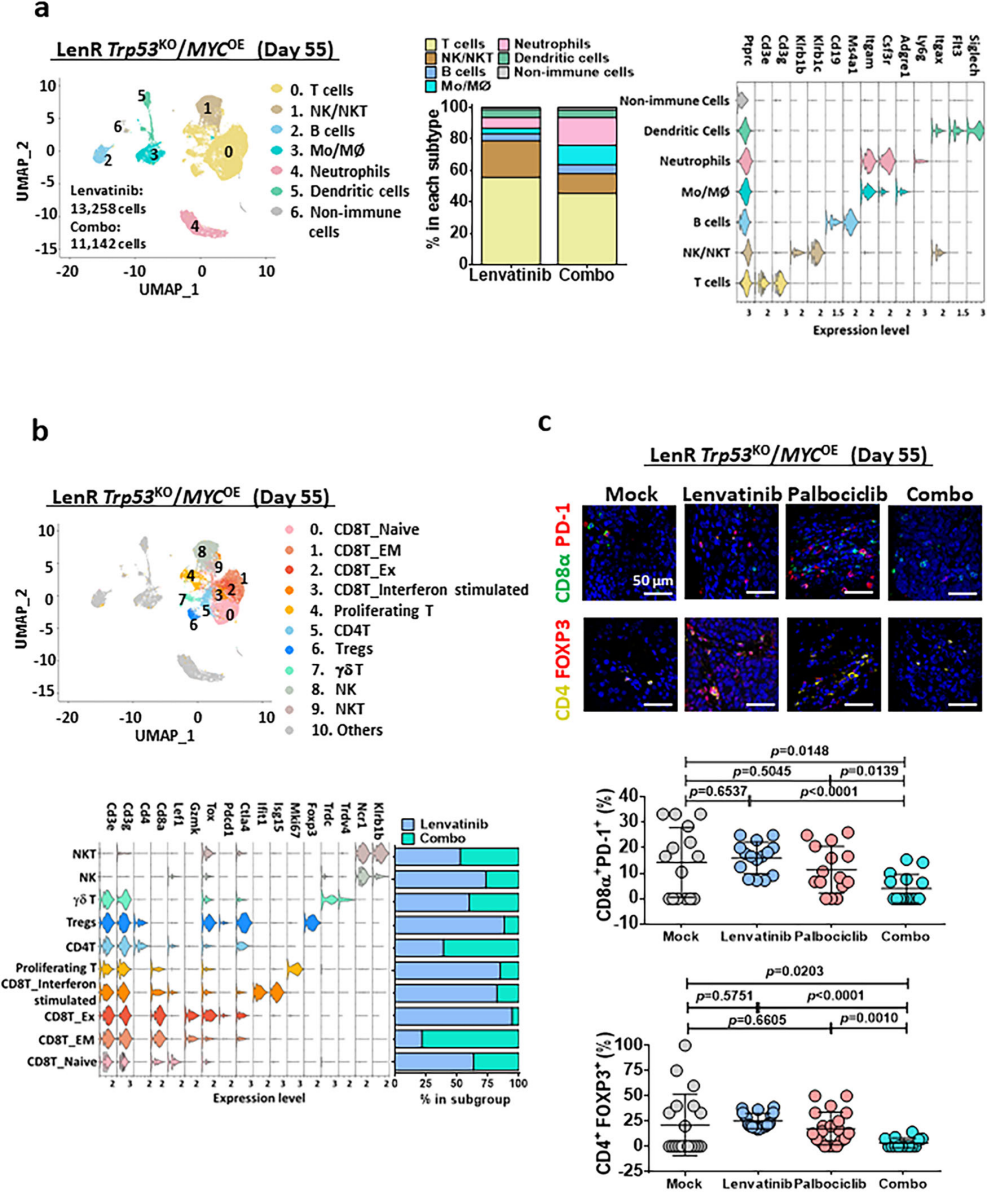
The effect of palbociclib/lenvatinib treatment in modulating the tumor micro-environment in the lenvatinib-resistant *Trp53*^KO^/*MYC*^OE^ immune-competent mouse model. **a** Uniform manifold approximation and projection (UMAP) plot showing 7 distinct clusters resulting from scRNA sequencing of sorted CD45^+^ cells derived from tumors harvested from lenvatinib or combo treatment. The percentage of major immune cell types in lenvatinib or combo group was calculated. Violin plots displayed the expression of key lineage-defining genes for the major subtypes of immune cells. **b** UMAP visualization of T cells and NK/NKT cells only. Violin plots exhibited the expression of the key subtype-defining genes. Percentage change in each subtype was shown and combo treatment led to a decrease in CD8-expressing exhausted T and Treg cells. **c** Multiplex IHC staining on exhausted CD8T and Treg cells were performed using combination of CD8α (green)/PD-1 (red) (*n* = 15 random views) or CD4 (yellow)/FOXP3 (red) (*n* = 14 random views in mock, *n* = 20 random views in lenvatinib and palbociclib while *n* = 14 random views in combo) in resected tumors from lenvatinib-resistant *Trp53*^KO^/*MYC*^OE^ (two-tailed *t* test). Scale bar = 50 µm. Number of positive cells was quantified using ImageJ. Data was presented as mean ± standard deviation. Source data are provided as a Source Data file.

Next, we further examined the therapeutic efficacy of palbociclib to sensitize lenvatinib-resistant HCC cells in an immunocompetent mouse model. HCC was induced in wild-type C57BL/6 J mice by overexpressing activated forms of c-myc-luc with PX330-sg-p53 along with sleeping beauty transposase by hydrodynamic injection. After injection of plasmids for 12 days, mice were treated with 30 mg/kg lenvatinib for 25 days to mimic the clinical situation in which lenvatinib-nonresponsive patients with HCC progress after lenvatinib treatment (Fig. 9a). Successful establishment of lenvatinib resistance was evidenced by the rebound of the signal intensity upon lenvatinib treatment (Fig. 9b). Such an increase in signal intensity was paralleled with induced expression of CDK6, but not CDK4, upon acquisition of the lenvatinib resistance (Supplementary Fig. 13a, b). Interestingly, pluripotency related marker OCT4 was also induced during the process (Supplementary Fig. 13a, b). At this point, the mice in groups of 7-8 were divided into 4 groups with the same treatment as shown in Fig. 9a. The efficacy of the combined drug treatment was evaluated by the liver weight-to-body weight ratio as well as signal intensity, and it was compared to the single-drug treatment groups. Treatment with palbociclib/lenvatinib led to the maximal suppression of tumor growth, indicating that palbociclib treatment synergizes with lenvatinib treatment and is effective against liver tumors in vivo (Fig. 9c–e). Similar to Fig. 8e, a decrease in β-catenin and PCNA was observed in the combination treatment group (Fig. 9f). Recently, β-catenin activation has been found to promote immune evasion in HCC^17^. Because we demonstrated the role of CDK6 in the activation of β-catenin in lenvatinib-resistant HCC cells, we hypothesized that palbociclib converts the immune-suppressive environment to an immune-inflamed environment. For this purpose, we extracted CD45^+^ cells from tumors treated with either lenvatinib or the combination and analyzed them by single-cell RNA (scRNA) sequencing. ScRNA sequencing demonstrated that the total populations of regulatory T cells (Tregs) were decreased upon combined treated. Echoed to this observation, the subpopulation of memory effector T cells was increased while exhausted CD8^+^ T cells were decreased (Fig. 10a, b). The scRNA sequencing data was validated by multiplex fluorescent IHC staining of exhausted CD8^+^ cells labeled by CD8α and PD-1, while Treg cells marked by CD4 and FOXP3 (Fig. 10c). These findings demonstrated a remodeling of immune landscape after combined treatment with palbociclib and lenvatinib which favors immune suppression against HCC tumors.

## Discussion

Thus far, the molecular mechanisms of acquired resistance to lenvatinib remain elusive. There are few reports showing the role of EGFR^18^, dual specificity phosphatase 9 (DUSP9)^19^ and integrin subunit beta 8 (ITGB8)^20^ in the regulation of lenvatinib resistance in HCC. Using a chemical biology approach, we identified CDK6 as a targetable resistance mechanism for lenvatinib in HCC. Echoed with this finding, we found significant upregulation of CDK6 in lenvatinib-resistant HCC patients when compared with sensitive group in seven cases of lenvatinib-treated HCC samples. The sample size of our study was limited due to the fact that lenvatinib was FDA-approved as a first-line treatment for HCC in 2018, which contrasts with the larger collection of HCC patient samples treated with sorafenib, which was approved for HCC treatment in 2007. Recently, CDK6 upregulation has been observed in multiple myeloma patients refractory to lenalidomide treatment^21^. The role of CDK6 in cancer remains controversial. CDK6 is overexpressed in lymphoma^22^, leukemia^23^, glioblastoma^24^, colorectal cancer^25^ and esophageal cancer^26^. However, CDK6 overexpression has been shown to reduce tumorigenesis in melanoma and B cell lymphoma^27,28^, while suppression of CDK6 leads to suppression of tumor cell growth in acute myeloid leukemia^29^, colorectal cancer^30^, and breast cancer^31^. To date, the clinical relevance and functional role of CDK6 in HCC remain poorly characterized. In the present study, we found that CDK6 was overexpressed at both the mRNA and protein levels with clinical significance.

CDK6 was reported to be crucial in the activation of hematopoietic and leukemic stem cells through direct transcriptional suppression of early growth response 1 (*Egr-1*)^32^. Subsequently, it has been reported that enforced CDK6 expression shortens the timing for the exit of cell cycle arrest, resulting in enhanced repopulation capacity^33^. Although these findings suggest a potential role for CDK6 in regulating CSCs, the molecular mechanism by which CDK6 regulates CSC properties is poorly understood. Whether CDK6 regulates liver CSCs also remains unexplored. By overexpression and knockdown approaches, we demonstrated that CDK6 is critically involved in the regulation of liver CSCs and lenvatinib resistance. Despite the high degree of homology between CDK4 and CDK6, increasing evidence has demonstrated the distinct role of CDK6^34^. Nonetheless, we cannot rule out the possibility that CDK4 may play a role in liver CSCs and lenvatinib resistance in a subset of HCC patients. As a serine/threonine protein kinase, CDK6 plays a crucial noncanonical role in diverse cellular processes, including inflammation^35^ and cellular differentiation^36^, by phosphorylating NPM1 and NF-κB. In addition, CDK6 also acts as a transcriptional regulator in a kinase-independent manner by interacting with STAT3, *Runx1,* and c-Jun^27,37,38^. Based on our in vitro data, we showed that CDK6 regulates liver CSCs and lenvatinib resistance in a kinase-dependent manner. Our analysis also revealed that CDK6 upregulation is mediated by the ERK/YAP1 signaling cascade. This result is in line with other study showing CDK6 is the direct target of YAP-TEAD transcriptional active complex, as evidenced by the ChIP assay showing the binding of YAP on CDK6 promotion^39^. To understand the molecular mechanism by which CDK6 regulates liver CSC properties and tumor behavior, it is crucial to identify its direct interacting partner. By immunoprecipitation assays, we demonstrated unprecedentedly that CDK6 directly binds and phosphorylates GSK3β, resulting in prevention of β-catenin degradation mediated by ubiquitination. Consistently, we found that β-catenin expression was increased in CDK6-overexpressing HCC cells compared to control cells. The effect of CDK6 on the activation of the Wnt/β-catenin signaling pathway was further confirmed by the localization and activation of β-catenin transcription. Since CDK6/GSK3β/β-catenin cascade was similarly observed in HCC cell lines with wild-type p53 and its mutated form, it suggests that this proposed mechanism may be independent of p53 status. This pathway is clinically relevant, as shown by an enrichment of the Wnt/β-catenin pathway in CDK6-overexpressing HCC patients in a cohort of HCC clinical samples. However, this result contradicts another report showing the negative regulatory role of CCND1-CDK6 on β-catenin in Wnt-stimulated mouse embryonic fibroblasts (MEFs)^40^. This discrepancy may be due to the difference in the cell system used in each study.

In the present study, we examined the therapeutic efficacy of palbociclib in combination with lenvatinib by oral gavage in a xenograft model derived from lenvatinib-resistant PLC/PRF/5 cells. After 21 days, palbociclib/lenvatinib treatment markedly reduced the tumor volume by 34.9% compared to the original volume at Day 0. Accompanied by this phenotypic change, we found that combination treatment led to a significant decrease in β-catenin expression. Interestingly, we found that the combination treatment also suppressed HCC proliferation as evidenced by the decrease in PCNA staining. Because β-catenin activation has been reported to promote immune evasion in HCC^17^, we examined whether palbociclib/lenvatinib treatment exerts a similar growth suppressive effect in a lenvatinib-refractory immune-competent mouse model by introducing C-MYC and sg*Trp53* along with SB by hydrodynamic tail vein injection. We found that palbociclib/lenvatinib led to maximal suppression of tumor growth with decreased expression of β-catenin and PCNA. By scRNA sequencing, we found a decreased population of Tregs in the combination treatment group compared to the lenvatinib-treated group, which was consistent with the clinical observation in palbociclib-treated metastatic breast cancer patients^41^. Consistently, increased memory effector T-cell and decreased exhausted T-cell infiltration were observed in the combination-treated group, which implicated the potential role of palbociclib in the modulation of T cells for immune cancer therapy. This result is line with previous report showing promotion of CD8 memory T cells upon palbociclib treatment^42^.

In conclusion, we demonstrated that CDK6 blockade impedes the lenvatinib-induced activation of the ERK/YAP/CDK6/β-catenin signaling cascade (Supplementary Fig. 14). In addition, palbociclib in combination with lenvatinib reshapes the immune tumor microenvironment which favors immune suppression against HCC. Targeting CDK6 with palbociclib in combination with lenvatinib may be an innovative and safe therapeutic strategy against HCC.

## Methods

### Study approval

Clinical HCC samples for tissue microarray were obtained by Profesor Jing-Ping Yun at the Sun Yat-sen University Cancer Centre in Guangzhou, China, with the approval by the Institutional Review Board for ethical review from the University (G-2022-105-01). Clinical samples for lenvatinib-sensitive and lenvatinib-resistant HCC tumors were obtained from Dr. Qian Qian Yu at Tongji Hospital, Tongji Medical College, Huazhong University of Science and Technology, Wuhan, China, with the approval by the Institutional Review Board for ethical review from the University (2019s1096). Informed consent was obtained from patients. License to conduct experiments on animals was obtained from the Department of Health, Hong Kong SAR. Approval to conduct animal work at the Hong Kong Polytechnic University was obtained from the Animal Subjects Ethics Sub-Committee.

### Plasmids and reagents

The CDK6-WT and CDK6-K43M mutant pLenti.6.2/V5-DEST^TM^ vectors were kind gifts from Dr. Claudia Scholl (National Center for Tumor Diseases and German Cancer Research Center (DKFZ)). LATS-Biosensor (NLuc-YAP15 (#107610) and 14-3-3-CLuc (#107611)) were obtained from Addgene. Lenvatinib was purchased from Selleckchem (in vitro experiments) and LC Laboratories (in vivo experiments). The CDK4/6 inhibitor, palbociclib, and the MEK inhibitor, U0126, were purchased from LC Laboratories. The YAP inhibitor, CA3, was purchased from Selleckchem. The PROTAC CDK6 degrader, CP-10, was purchased from MedChemExpress, while BSJ-03-123 was purchased from Tocris Bioscience. MG132 was purchased from Calbiochem.

### Cell lines and cell culture

The following cells lines were maintained in Dulbecco’s Modified Eagle Medium (DMEM) with high glucose and L-glutamine (Gibco, Invitrogen) supplemented with 10% heat-inactivated fetal bovine serum (FBS) (Gibco, Invitrogen), 100 mg/mL penicillin G and 50 µg/mL streptomycin (Gibco, Invitrogen) at 37 °C in a humidified chamber containing 5% CO_2_: MHCC-97L and MHCC-97H human HCC cell lines (Liver Cancer Institute, Fudan University); Hep3B, HepG2, and SNU-182 human HCC cell lines (America Type Culture Collection, Manassas, VA, USA); Huh7 and PLC/PRF/5 human HCC cell lines (Japan Cancer Research Bank) and 293FT (Invitrogen, Thermo Fisher Scientific, Waltham, USA). The culture medium was refreshed every 2 days. All cell lines used in this study were obtained between 2013 and 2016, and they were authenticated by morphological observation and STR DNA analysis with PowerPlex® 16HS kit (Promega) as well as tested for the absence of mycoplasma contamination (MycoAlert, Lonza). Cells were used within 20 passages after thawing.

### Establishment of lenvatinib-resistant HCC cells

Lenvatinib-resistant clones of PLC/PRF/5 and Huh7 cells were established by subjecting HCC cells to continuous administration of gradually increasing lenvatinib concentrations and were trained up to 30 µM for up to 6 months. The same volume of DMSO was added to the cells as mock controls during the establishment of these resistant cells. The established lenvatinib-resistant clones were maintained in 10 µM lenvatinib.

### Collection of lenvatinib-sensitive and lenvatinib-resistant-HCC tumor samples

Seven archived paraffin-embedded pathological specimens from primary HCC patients were collected along with complete clinical and pathological data at the Tongji Hospital, Tongji Medical College, Huazhong University of Science and Technology, Wuhan, China. All samples were anonymous. Participants were Chinese male aged from 43 to 57. This study was approved by the Institutional Review Board for ethical review from the University (2019s1096). The procurement of all clinical information has received consent from patients. The clinicopathological features of the patients were summarized in Supplementary Table 1.

### Tumor response assessment

Evaluation of tumor response according to the RECIST 1.1 was defined as follows: complete response (CR) is the disappearance of all target lesions; partial response (PR) is at least a 30% decrease in the sum of the diameters of the target lesions; progressive disease (PD) is at least a 20% increase in the sum of the longest diameters of target lesions or the appearance of one or more new lesions; stable disease (SD) is the lesion diameter decreased by <30% to increased by <20%. According to mRECIST, CR is defined as the disappearance of any intratumoural arterial enhancement in all target lesions; PR is at least a 30% decrease in the sum of diameters of viable target lesions, taking as reference the baseline sum of the diameters of target lesions; PD is an increase of at least 20% in the sum of the diameters of viable target lesions, taking as reference the smallest sum of the diameters of viable target lesions recorded since the treatment started; SD is any cases that do not qualify for either PR or PD. Objective response (OR) included both CR and PR, and disease control included CR, PR and SD.

### Tissue microarray of HCC samples

51 archived paraffin-embedded pathological specimens from primary HCC patients were collected along with complete clinical and pathological data at the Sun Yat-sen University Cancer Center. All samples were anonymous. This study was approved by the Institute Research Medical Ethics Committee (G-2022-105-01). Among the 51 samples collected, 47 participants were male while 4 were female. Median age of the participants was 52. None of the patients had received radiotherapy or chemotherapy before surgery. The clinicopathological features of the patients were summarized in Supplementary Table 3.

### Long-term clonogenic assay

HCC cells were seeded in six-well plates at density of 1 × 10^3^–5  × 10^3^ 24 hours prior to treatment with either DMSO or lenvatinib for 10 to 14 days depending on growth rate. Colonies were fixed with 4% paraformaldehyde and stained with 0.1% crystal violet. Stained colonies were washed with water, air-dried, and visualized by using Chemidoc imaging system (Bio-Rad). The colonies were quantified using ImageJ.

### MTT assay

HCC cells were seeded on 96-well plates 24 hours prior to addition of indicated drugs (palbociclib: 1–10 µM; lenvatinib: 5–20 µM; U0126: 0.1–100 µM; CA3: 0.1–200 µM) or PROTAC CDK6 degraders (CP-10: 1–1000 nM; BSJ-03-123: 0.01–2 µM). After 48–144 hours, 3-(4,5-dimethylthiazol-2-yl)-2,5-diphenyl tetrazolium bromide (MTT) dye, at a concentration of 5 mg/ml (Invitrogen), was added and the plates were incubated for 3 hours in a moist chamber at 37 °C. Optical density was determined by eluting the dye with isopropanol supplemented with hydrochloric acid. The absorbance was measured at 570 nm.

### Knockdown of CDK6 and CTNNB1

To establish CDK6-knockdown clones, lentiviral particles were generated by co-transfecting 293FT cells with shCDK6 from MISSION® shRNA human gene set family bacterial glycerol stock (#SH0111, Sigma Aldrich), Clone IDs: TRCN0000039743 and TRCN0000039744, or non-target control (NTC) plasmids and packaging plasmid mix. Viral supernatant was collected for infection of MHCC-97L and Hep3B. Plasmids for silencing CTNNB1 were cloned using pLKO.1-puro lentiviral vector. Viral supernatant generated was used for infection of mock and lenR PLC/PRF/5 and Huh7. The stable knockdown cell lines were generated by puromycin selection. The shRNA sequences were listed in Supplementary Table 4.

### Overexpression of CDK6 by CRISPR activation

Gene activation of CDK6 was performed using Edit-R transcriptional activation system (Dharmacon). PLC/PRF/5 cells were engineered for stably expression of dCas9-VP64-p65-Rta (dCas9-VPR) using blasticidin. Subsequent transduction of lentiviral CRISPR activation (CRISPRa) sgRNA targeting the promoter region of CDK6 was performed in dCas9-VPR stable cells. CDK6 stable expressing cell line was generated after puromycin selection. The sgRNA sequences were listed in Supplementary Table 4.

### Limiting dilution assay

PLC/PRF/5, MHCC-97L and Hep3B cells were cultured in a 96-well plate coated with poly (2-hydroxyethyl methacrylate) (polyHEMA, Sigma Aldrich) per condition with 200 μl serum-free 0.25% methylcellulose (Sigma Aldrich) DMEM/F12 medium supplemented with 4 µg/mL insulin (Sigma Aldrich) and B27 (Invitrogen) for PLC/PRF/5 and MHCC-97L, while 20 ng/mL EGF (Sigma Aldrich) and 20 ng/mL basic FGF (Invitrogen) were also used for Hep3B. Sphere formation was scored after 8–12 days under a phase contrast microscope. The frequency of sphere-forming cell was calculated accordingly using an extreme limiting dilution algorithm (ELDA) (http://bioinf.wehi.edu.au/software/elda/)^43^.

### Migration and invasion assays

Migration assays were performed using polycarbonate membrane transwell inserts with pore size of 8 µm (Millipore). Invasion assays were performed using self-coated Matrigel (BD Biosciences) transwell inserts. HCC cells in serum-free medium were seeded in upper chamber while the lower chamber was supplemented with 10% FBS medium as chemoattractant. Cells were incubated in humidified incubation at 37 °C for 48 hours. The transwell membranes were fixed with methanol and stained with 1% crystal violet. The membranes were cleaned and air-dried. Photographs of five randomly selected fields of the fixed cells were captured and the cells were counted using ImageJ.

### In-gel fluorescence visualization of XO44-labeled kinases

The XO44 probe (Sigma, PF-6808472) was added to live PLC/PRF/5 cells and Huh7 cells at a final concentration of 2 µM followed by incubation for 30 minutes at 37 °C. Cell pellets were collected by centrifugation at 300 × *g* at 4 °C and resuspended in lysis buffer containing 100 mM HEPES, 150 mM NaCl, 0.1% NP-40, 1 mM PMSF, and 1× complete EDTA-free protease inhibitor cocktail (Roche). Cellular debris was removed by centrifugation at 15,000 × *g* for 30 minutes at 4 °C. Cell lysates were used for the click reaction via Cu(I)-catalyzed azide-alkyne cycloaddition (CuAAC). Click chemistry was initiated by sequential addition of the following to each lysate: 1% SDS, 100 µM TAMRA-azide or biotin-azide, 1 mM TCEP, 100 µM TBTA, and 1 mM CuSO. After 1.5 hours of incubation at room temperature, the reactions were quenched by acetone precipitation. The precipitated proteins were pelleted by centrifugation at 1000 × *g* at 4 °C for 10 minutes, and the pellets were resuspended in sample buffer and resolved by 12% SDS-PAGE. Gels were scanned by a Typhoon 9400 system (Typhoon Imaging System, GE) under the Cy3/Cy5 channel for fluorescence bands and protein marker visualization. The gels were then stained with Coomassie blue to show the protein loading amount.

### Liquid Chromatography with tandem mass spectrometry (LC-MS/MS) analysis

LC-MS/MS analyses were performed on an Exploris 480 Orbitrap mass spectrometer (Thermo Fisher Scientific) coupled with an UltiMate 3000 UPLC system (Thermo Fisher Scientific). A C18 analytical column (75 μm × 250 mm, 1.6 μm, 120 Å) (Aurora, Ion opticks) was employed for LC separation. Mobile phases A and B consisted of 0.1% FA in water and 0.1% FA in 80% ACN, respectively. The following setup was used for LC separation with a flow rate of 300 nL/minute: mobile phase B at 8% for 120 minutes; mobile phase B was increased to 35% at 85 minutes and 90% at 95 minutes and held for 13 minutes; and mobile phase B was returned to 8% at 110 minutes and maintained until 120 minutes. Data was collected in data-dependent acquisition (DDA) mode. The precursor ions with a charge state of 2+ or higher were fragmented by higher-energy collisional dissociation (HCD). The MS1 Orbitrap resolution was set at 120,000, and the MS1 AGC target was set at 3 × 10^5^. The MS2 Orbitrap resolution was set at 30,000, and the MS2 AGC target and the maximum injection time were set at 1 × 10^5^ and 50 ms, respectively.

### MS data analysis of DDA data

The acquired label-free DDA data were searched against the Homo sapiens UniProt database (Version June 2020, 20368 entries) using the SEQUEST algorithm (Proteome Discoverer 2.4, Thermo Fisher Scientific). The precursor ion mass tolerance and fragmentation tolerance were set as 10 ppm and 0.02 Da for the database search, respectively. The maximum number of modifications per peptide was three. Carbamidomethylation on cysteine was set as a fixed modification, and oxidation on methionine and protein N-terminal acetylation were set as variable modifications. The enzyme was specified as trypsin with two missed cleavages allowed. The false discovery rate for peptide matches and proteins was set as 1%. The proteomics data generated have been deposited in proteomeXchange database under accession code PXD038152.

### Validation of CDK6 using a parallel reaction monitoring (PRM)-based assay

The same equipment and LC gradient were applied for PRM analyses. The PRM acquisition method was directly developed based on DDA data. The DDA data were imported into Skyline to recognize a unique peptide of CDK6. The relative information, including precursor *m/z*, charge, and retention time window of the selected peptide, was exported from Skyline into Xcalibur software to edit the PRM method. The targeted MS1 parameters were as follows: resolution, 120,000; AGC target, 3.0 × 10^5^; and maximum injection time, 100 ms. PRM scanning was performed at 60,000 resolution, 1 × 10^5^ AGC target, and a 1.0 *m/z* isolation window. After PRM data acquisition, the data were imported into Skyline for analysis. To obtain reliable identification and quantification, idotp and dotp should be above 0.70. The top three product ions were summed to represent the peptide abundance.

### STRING analysis

According to proteomics data, enhanced kinase with a fold-change greater than 2 (fold change (FC) = Lenvatinib-Resistant/Mock) from PLC/PRF/5 and Huh7 cells was input into the STRING platform (https://string-db.org/) for protein-protein interaction analysis.

### Phosphoproteome analysis

Phosphopeptide enrichment was performed using TiO2 for TMT6plex-based quantitative proteomics. LC-MS/MS analyses were performed on Orbitrap Fusion Lumos mass spectrometer (Thermo Fisher Scientific) coupled with an UltiMate 3000 UPLC system (Thermo Fisher Scientific). A RSLC C18 analytical column (75 μm × 250 mm, 2.0 μm, 100 Å) (Thermo Fisher Scientific) was employed for LC separation. TMT data were searched against the Homo sapiens UniProt database (Version June 2020, 20368 entries) using the SEQUEST algorithm (Proteome Discoverer 2.4, Thermo Fisher Scientific). Carbamidomethylation of cysteine residues was set as a static modification. TMT tags on lysine residues and peptide N-terminal, phosphorylation of serine (S)/threonine (T)/tyrosine (Y), and oxidation of methionine residues were set as variable modifications. The proteomics data generated have been deposited in proteomeXchange database under accession code PXD038152.

### LATS-Biosensor (LATS-BS) luciferase assay

LATS kinase activity was monitored by LATS-BS consisting of two vectors: NLuc-YAP15 (#107610, Addgene) and 14-3-3-CLuc (#107611, Addgene)^15^. Lenvatinib-resistant PLC/PRF/5 and Huh7 were transfected with LATS-BS and pRL-CMV Renilla luciferase (Promega) for normalization of transfection efficiency using Lipofectamine 2000. Treatment of 10 µM U0126 or solvent control (DMSO) for 4 hours was done at 48 hour post transfection. The cells were collected and luciferase signal was measured using Dual-luciferase report assay system (#E1910, Promega). Luciferase fold change was calculated according to the method described^15^.

### Annexin-V apoptosis assay

Cells were stained by FITC-conjugated Annexin-V (BioVision) and propidium iodide (PI) (Invitrogen) in Annexin-V binding buffer (BD Biosciences) at room temperature for 30 mins. Apoptosis percentage was determined using BD Accuri C6 flow cytometer and FACSDiva software (BD Biosciences).

### Isolation of CD45^+^ cells from tumors

Mouse tumor tissue samples were harvested from in vivo experiments were processed for single-cell isolation. Tumors were cut into small pieces in ice-cold perfusion buffer (25 mM HEPES pH7.4, 0.5 mM EDTA in PBS) and transferred to gentleMACS C tube (Miltenyi Biotec GmbH) with four rounds of dissociation in digestion buffer with 0.5 mg/ml Collagenase IV (Invitrogen) and 0.1 mg/ml DNaseI (Roche) using gentleMACS dissociator (Miltenyi) according to instructions provided by the manufacturer. The cell suspensions were passed through 70 µm cell strainers prior to loading to percoll gradient mix to create the gradient. After centrifugation, the pellets were subjected to red blood cell lysis (Sigma Aldrich) and resuspended in FACS buffer (2% FBS PBS). Isolated cells were stained with anti-CD45-APC antibody (#55984, BD Biosciences) for 45 minutes on ice. LIVE/DEAD Fixable Near-IR Dead Cell Stain Kit (Thermo Scientific) was used for exclusion of dead cells. Isolated cells were analyzed and the live CD45^+^ subpopulation was sorted on a BD FACSAriaIII cell sorter (BD Biosciences).

### Single-Cell RNA (scRNA) sequencing

The sorted live CD45^+^ cells stored in ice-cold FACS buffer were immediately processed according to manufacturer’s instructions by encapsulation into single-cell gel beads (GEMs) and unique molecular identifier (UMI) barcoding using the Chromium Next GEM 3’ v3 chemistry Single-Cell kit (10X Genomics, CA, USA) at The Beijing Genomics Institute. Libraries were prepared and sequenced on a NovaSeq platform (Illumina, San Diego, USA). The scRNA sequencing data generated by this study has been deposited to GEO database under accession number: GSE218010.

### Processing and quality control of scRNA sequencing data

Initial data demultiplexing, read alignment, UMI counting, and annotation of the raw read data were performed using the Cell Ranger Single-Cell software version 5.0.1 (10X Genomics). Raw sequencing reads of the two samples (Lenvatinib and Combo) were mapped to the mouse genome (mm10) and were analyzed with the Seurat R package version 4.1.1. The cells that had unique feature counts over 6000 or 25% mitochondrial counts, were filtered. The gene expression data was normalized and variance-stabilized using sctransform function and glmGamPoi improvement. Datasets of two samples were merged as Seurat object. Major immune cell clusters projected in two-dimensional UMAP representation were annotated to known cell lineages using both published marker genes^44^ and SingleR package^45^ with reference to Immunological Genomic Project (ImmGen) database^46^. CD8T-cell subtypes prediction was performed using TILPRED^47,48^.

### Flow cytometric analysis

Cells were stained by Phycoerythrin (PE)-conjugated-conjugated CD47, CD90, EpCAM (#536046, #555596, #347198, BD Biosciences) and CD133 (#130-120-059, Miltenyi Biotec) antibodies in PBS with 2% FBS at 4 °C for 30–60 minutes. Isotype-matched mouse immunoglobulins served as controls. Samples were analyzed using BD Accuri C6 flow cytometer and FACSDiva software (BD Biosciences).

### Western blot analysis

Whole cell lysates were extracted using either NETN buffer supplemented with protease inhibitor cocktail or direct lysis. Protein lysate was separated by SDS-polyacrylamide gel electrophoresis (SDS-PAGE) and transferred to polyvinylidene difluoride membrane (Millipore) for western blot analyses. Primary antibodies against CDK6, CDK4 [EPR2513Y], MAP3K7 (TAK1) [EPR5984] (1:1000, ab151247, ab68266, ab109526, Abcam), p-GSK3β (Ser9) (D3A4), β-catenin (D10A8), p44/42 MAPK, p-p44/42 MAPK, YAP (D8H1X), pYAP(Ser127) (D9W2I), SOX2 (D6D9), RPS6KA4 (MSK2) (D41A4) (1:1000, #9322 S, #8480 S, #9102 S, #9101 S, #14074 S, #13008, #3579, #3679, Cell Signalling Technology), GSK3β (clone 7) (1:1000, #610202, clone 7, BD Transduction Laboratories), HA (1C1D2) (1:1000, #66061-1-Ig-AP, Proteintech), OCT4 (C-10) (1:1000, sc5279, Santa Cruz) and β-ACTIN (AC-74) (1:5000, #A5316, Sigma Aldrich) were incubated at 4 °C overnight. After washing, the membrane was incubated with horseradish peroxidase-conjugated anti-mouse or rabbit antibody (GE HealthCare). The signals were visualized using the enhanced chemiluminescence method. Blot images were quantified by densitometry using ImageJ software.

### β-catenin TCF binding luciferase reporter assay

β-catenin activity was examined using luciferase reporter assay of TCF/LEF-dependent transcription (TOP/FOPFLASH reporter assay). Either firefly luciferase pSuper8XTOPflash or pSuper8XFOPflash constructs (gifts from Dr. Moon R, University of Washington, USA), together with Renilla luciferase construct pRL-CMV (Promega, Madison, WI, USA) for normalization of transfection efficiency, were transfected using Lipofectamine® 2000. Luciferase activities were assayed using Dual-Luciferase® Reporter Assay System (Promega) according to manufacturer’s protocol.

### Immunofluorescence staining

HCC cells were seeded on glass coverslips, fixed in 4% paraformaldehyde (PFA) in PBS for 10 minutes and permeablized with 0.1% Triton-X100 for 10 minutes. Cells were blocked with 5% bovine serum albumin (BSA) in TBST for one hour. Anti-β-catenin (D10A8) (1:100, #8480 S, Cell Signaling Technology) was applied and stained at 4 °C overnight. Secondary antibody of goat anti-rabbit conjugated with Alexa Fluor^TM^ 488 (1:500, #A-11008, Thermo Scientific) was applied on the next day for an hour at room temperature. Slides were counterstained with DAPI for nuclei, mounted and subjected to Leica TCS SPE confocal microscope examination.

### Multiplexed fluorescent immunohistochemistry in HCC specimens and resected mouse tumor

The tyramide signal amplification-based method was used for staining multiple targets in HCC paraffin embedding specimens with Opal 4-Color Manual IHC Kit (NEL810001KT, Akoya Biosciences). Sections were deparaffinized in xylene and rehydrated in decreasing graded alcohols and distilled water. Slides were processed for antigen retrieval by a standard inverter microwave heating technique with either diluted 50× Envision FLEX Target Retrieval Buffer (pH9.0, K8004, Dako) for CDK6, YAP, p44/42 MAPK or 10× AR6 sodium citrate buffer for GSK3β (pH6.0, AR600250ML, Akoya BioSciences) for 15 minutes. Endogenous peroxidase activities were quenched using 3% hydrogen peroxide for 10 minutes at room temperature. The sections were immersed in blocking/antibody diluent (ARD1001EA, Akoya Biosciences) for 30 minutes at room temperature. Specimens were incubated with primary antibodies (CDK6 [EPR4515]: 1:200, ab124821, Abcam; GSK3β (H-76): 1:100, sc9166, Santa Cruz; YAP (D8H1X) & p44/42 MAPK: 1:100, #14074 S, #9102 S; CD4 (D7D2Z), CD8α (D4W2Z), PD-1 (D7D5W): 1:500, #25229 S, #98941 S, #84651 S, Cell Signaling Technology; FOXP3 (5H10L18): 1:100, #700914, Thermo Scientific). The sections were then washed thoroughly and incubated with Opal polymer HRP Mouse+Rabbit (ARH1001EA, Akoya Biosciences) for 30 minutes at room temperature. Followed by a brief wash with 1xTBST, Opal fluorophore (1:100) was applied for CDK6 and CD8 (Opal 520), GSK3β, YAP and CD4 (Opal 570), p44/42 MAPK, PD-1 and FOXP3 (Opal 690) for 15 minutes at room temperature. A final stripping step was performed with diluted 10× AR6 sodium citrate buffer in the microwave oven for 15 minutes. The section slides were cooled down, counterstained with DAPI solution (1:1000) and mounted for examination using Leica TCS SPE confocal microscope.

### Immunoprecipitation

The protein lysate was incubated with anti-CDK6 antibody (D4S8S) (#13331, Cell Signaling Technology) or anti-GSK3β (clone 7) antibody (#610202, BD Transduction Laboratories) together with Protein A agarose beads (#9863, Cell Signaling Technology) at 4 °C overnight with gentle agitation. Normal rabbit or mouse IgG served as control. The immunoprecipitates were eluted by boiling in 2× SDS loading buffer for 10 minutes and then subjected to SDS-PAGE and immunoblotting analyses.

### In vitro kinase assay

Kinase assay was performed using recombinant human CDK6 (rhCDK6) and GSK3β protein (rhGSK3β) as substrate (TP327978 & TP300468, Origene). In 50 µl 1× kinase buffer (#9802, Cell Signaling Technology), 500 ng of rhCDK6 was incubated with 10 ng of rhGSK3β with 0.2 mM ATP (#9804, Cell Signaling Technology) at 30 °C for 30 minutes. The reaction was terminated with 10 µl 6× SDS buffer at 95 °C for 10 minutes. The kinase activity of CDK6 reacting on GSK3β was determined by phosphorylation of GSK3β at serine 9 (p-GSK3β (Ser9) (D3A4), 1:1000, #9322 S, Cell Signaling Technology) using western blot analysis.

### RNA extraction and quantitative PCR (qRT-PCR) analysis

Total RNA was isolated using TRIzol reagent according to the manufacturer’s protocol (Invitrogen). Complementary DNA (cDNA) was synthesized using PrimeScript RT Reagent Kit (Takara Bio, Shiga, Japan) according to the manufacturer’s instructions and then subjected to qPCR with BrightGreen 2× qPCR Master mix (Applied Biological Materials Inc, Richmond, Canada) using QuantStudio 7 Flex Read Time PCR System (Applied Biosystems, Foster City, California, US) with primers specific to the sequences of genes of interest which were provided in Supplementary Table 5. Relative expression differences were calculated using 2^−ΔΔCT^method with reference to HPRT.

### Ubiquitination assay

The HA-tagged ubiquitin-expressing construct, pcDNA3.1-HA-(Ub)_8_, was transfected into HCC cells and treated with 10 µM MG132 (Calbiochem) for 8 hours at 48-hour post transfection. The cells were lysed with RIPA buffer (#9806, Cell Signaling Technology) supplemented with protease inhibitor cocktail and phosphatase inhibitor (Roche). The protein lysate was immunoprecipitated overnight with anti-β-catenin (D10A8) antibody (#8480 S, Cell Signaling Technology) antibody at 4 °C with gentle rotation overnight, followed by Protein A agarose incubation (Cell Signaling Technology) for 8 hours at 4 °C with gentle agitation. Immunoprecipitates were washed thrice with 1% TritonX-100-PBS, eluted by boiling in 2× SDS loading buffer for 10 minutes, and subjected to immunoblotting analyses using primary antibodies against HA (1F5C6) (1:1000, 66006-1-Ig, Proteintech) and β-catenin (D10A8) (1:1000, #8480 S, Cell Signaling Technology).

### Immunohistochemical analysis of HCC specimens and resected mouse tumors

Sections were deparaffinized in xylene and rehydrated in graded alcohols and distilled water. Slides were processed for antigen retrieval by a standard microwave heating technique in Tris-EDTA buffer. Endogenous peroxidase activities were quenched using 3% hydrogen peroxide. The sections were immersed in serum-free-protein block solution (DAKO). Specimens were subsequently incubated with primary antibodies (CDK6 [EPR4515]: 1:200, ab124821; PCNA (PC10): 1:5000, ab29, Abcam; β-catenin (D10A8): 1:100, #8480 S; CDK4 (D9G3E): 1:200, #12790, Cell Signaling Technology). The sections were then washed thoroughly and incubated with anti-rabbit Envision^TM^ HRP-conjugated secondary antibody (DAKO). Positive signals were visualized using Liquid DAB+ Substrate-Chromogen System (DAKO). Sections were counterstained with Mayer’s hematoxylin followed by examination using light microscope. For quantitation of CDK6 expression, the stained sections were assessed with no prior knowledge of the clinicopathological data for the patients. Each specimen was individually scored from 1 to 4 in terms of percentage (P) of expression, ≤10% stained positive, ≤25% stained positive, <50% stained positive and ≥50 stained positive, respectively. For intensity (I), each specimen was individually score from 1 to 3, 1 represents weak; 2 represents moderate; and 3 represents strong. Quick score was obtained by multiplying the percentage of positive cells (P) by the intensity (I). Formula: Q = P × I; Maximum = 12. The specimens with score <6 belong to the “low expression” group while those ≥6 belong to “high expression”. The expression of CDK4, PCNA and β-catenin in mouse tumor tissues were quantified using ImageJ software.

### Animal experiments

All mice were housed in university facility in 12 hours light/dark cycle (8:00–20:00 light, 20:00–8:00 dark), with controlled room temperature (23 ± 2 °C) and humidity (30–70%), in groups according to stocking density as recommended.

In vivo evaluation of tumorigenicity was performed with male NOD/SCID mice of age 4–6-week-old by induction of tumor xenografts. Cells were suspended in 1:1 culture medium and BD Matrigel Matrix (BD Biosciences) and subcutaneously injected into the flanks of the NOD/SCID mice, which were kept under observation. Briefly, each mouse received two injections of cells in both flanks, and cells from each experimental group (NTC vs shCDK6; sgCTRL vs sgCDK6) were injected into different mice. Tumors were harvested at the end of the experiment for documentation. Tumor-initiating cell frequency was calculated using extreme limiting dilution analysis (ELDA) software^42^. No specific randomization method was used. Sample size of animals was chosen based on significant p values.

For establishment of a lenvatinib-resistant *Trp53*^KO^/*MYC*^OE^ HCC mouse model, six- to eight-week-old male wild-type C57BL/6 J mice were used for the HCC mouse model. Plasmids (15 μg) encoding human MYC (C-MYC) and sg*Trp53* along with sleeping beauty (SB) transposase in a ratio of 25:1 were diluted in 2 mL of saline (0.9% NaCl), filtered through a 0.22 μm filter and injected into the lateral tail vein of C57BL/6 J mice in 5–7 seconds. Upon injection of plasmids for 12 days, mice were treated with 30 mg/kg lenvatinib for 25 days. Successful establishment of lenvatinib resistance was evaluated by the IVIS imaging *s*ystem (Perkin-Elmer).

For in vivo drug treatment assay, a total of 1 × 10^5^ lenvatinib-resistant PLC/PRF/5 cells were injected into the flanks of male BALB/c nude mice of age 4-6 week-old. Once the tumors were established and reached ~6 mm × 6 mm (length × width), the mice were randomly divided into four groups as follows: (i) mock (0.5% methylcellulose in saline); (ii) lenvatinib (30 mg/kg); (iii) palbociclib (100 mg/kg); and (iv) the combined treatment of palbociclib and lenvatinib. Palbociclib was resuspended in 0.5% methylcellulose in saline. Lenvatinib was resuspended in water. The mice were given lenvatinib and palbociclib orally on a daily basis. The tumor volume and body weight were measured every three days. The tumor volume was calculated using the following formula: volume (cm^3^)  =  length × width^2^ × 0.5. The mice were treated for 21 days before sacrifice, at which point the tumors were harvested for analysis.

The study protocol was approved by and performed in accordance with the guidelines for the Use of Live Animals in Teaching and Research at Hong Kong Polytechnic University. The study protocol was approved by and performed in accordance with the guidelines for the Use of Live Animals in Teaching and Research at Hong Kong Polytechnic University. For subcutaneous tumor model, the tumor volumes did not exceed 10% of normal body weight or 1.3 cm in diameter. Mice were sacrificed if the percentage of body weight loss is greater than 20%. For HTVI model, mice were euthanasia if the percentage of body weight gain is greater than 10% or weight loss is greater than 20%. The maximal tumor size/burden was permitted by study protocol of the Hong Kong Polytechnic University. The maximal tumor size/burden was not exceeded in all experiments.

### Statistical analysis

The statistical significance of the results obtained from limiting dilution assay, flow cytometric analysis, migration and invasion assay, qRT-PCR, immunohistochemical and immunofluorescence staining, in vivo tumor growth and volume were determined by *t* test or Mann–Whitney *U* test wherever appropriate using GraphPad Prism (GraphPad Software, San Diego, CA). The results are presented as means and standard deviations, and *p* values <0.05 were considered statistically significant. A data point was excluded if it deviated from the mean by more than three standard deviations. Investigators were not blinded to the group allocation during the experiment or when assessing the outcome in all experiments, including animal experiments. There was no estimate of variation within each group of data. The variance was similar between the groups that were being statistically compared. Kaplan–Meier survival analysis was used to analyze disease-free survival, and a log-rank test was used to determine the statistical significance.

### Reporting summary

Further information on research design is available in the Nature Portfolio Reporting Summary linked to this article.

### Supplementary information

Supplementary Information

Reporting Summary

### Source data

Source Data

## Data Availability

The scRNA sequencing data generated by this study has been deposited to GEO database under accession number (GSE218010). The proteomics data generated have been deposited in proteomeXchange database under accession code PXD038152. The mRNA expression of *CDK6*, *YAP1,* and *ERK2* in human HCC patients were retrieved from transcriptome sequencing data available at the Cancer Genome Atlas (TCGA) (cBioportal for Cancer Genomics: https://www.cbioportal.org/). Transcriptome profiling data of human normal and cirrhotic livers, early to advanced stage of HCC dataset (GSE25097)^10^ was directly accessed through GEO website (NCBI). The remaining data are available within the Article, Supplementary Information, or Source Data file. Source data are provided with this paper.
