# Supplementary material for: Broad-spectrum kinome profiling identifies CDK6 upregulation as a driver of lenvatinib resistance in hepatocellular carcinoma
Source: Nat Commun. 2023 Oct 23;14:6699. doi: 10.1038/s41467-023-42360-w (PMC10593849; doi:10.1038/s41467-023-42360-w)
Supplement: Supplementary file 1 — Supplementary Information [file 41467_2023_42360_MOESM1_ESM.pdf]

Supplementary Information: Broad-spectrum kinome profiling identifies CDK6 upregulation as a driver of lenvatinib resistance in hepatocellular carcinoma

Table of content:

Supplementary Figure 1-14

Supplementary Table 1. Clinicopathological features of the lenvatinib-sensitive and resistant HCC patients.

Supplementary Table 2. Effect of CDK6 alterations on tumorigenicity.

Supplementary Table 3. The clinicopathological features of 51 HCC patients.

Supplementary Table 4. shRNA and sgRNA sequences.

Supplementary Table 5. Primer sequences for qRT-PCR.

Supplementary References

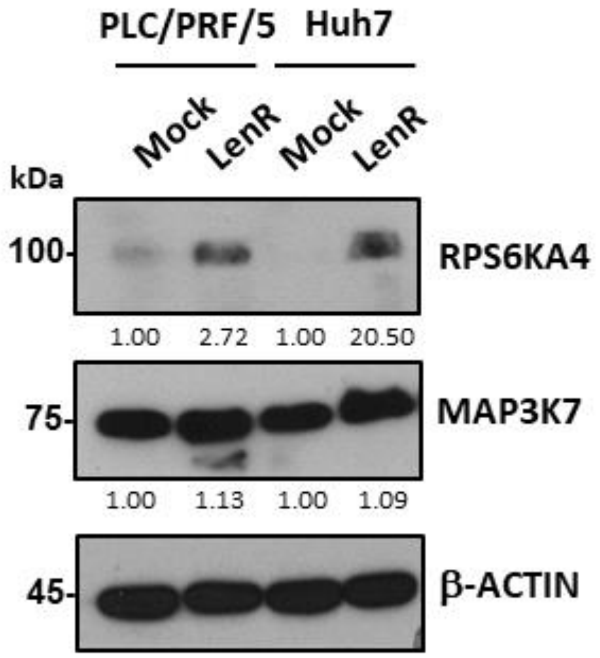

Supplementary Fig. 1. **Expression of RPS6KA4 and MAP3K7 in lenvatinib-resistant HCC cell lines.** By western blot analysis, protein expression of RPS6KA4 and MAP3K7 was upregulated in lenvatinib-resistant (LenR) cells when compared with mock counterparts. Expression of RPS6KA4 and MAP3K7 was normalized to β-actin and expressed in fold change relative to corresponding mock counterparts (*n*=2 independent experiments). Source data are provided as a Source Data file.

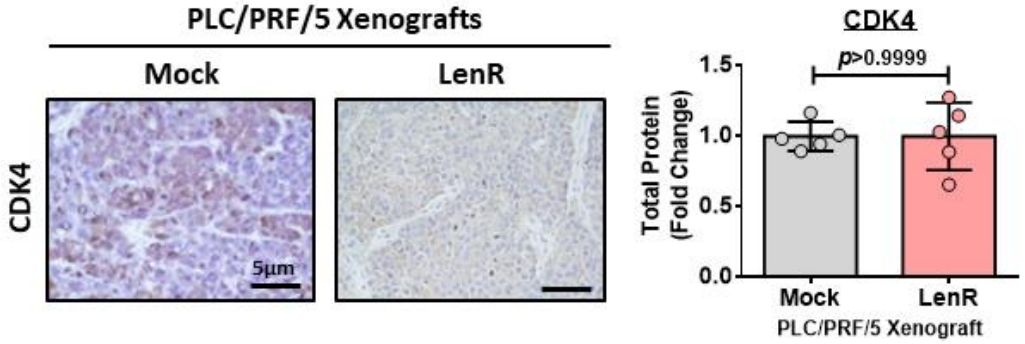

Supplementary Fig. 2. Immunohistochemical staining of CDK4 in mock and LenR PLC/PRF/5 xenografts. No significant change of CDK4 expression was observed in LenR PLC/PRF/5 xenograft when compared to the mock (n=5, two-tailed *t* test). Scale bar=5 µm. Data was presented as mean ± standard deviation. Source data are provided as a Source Data file.

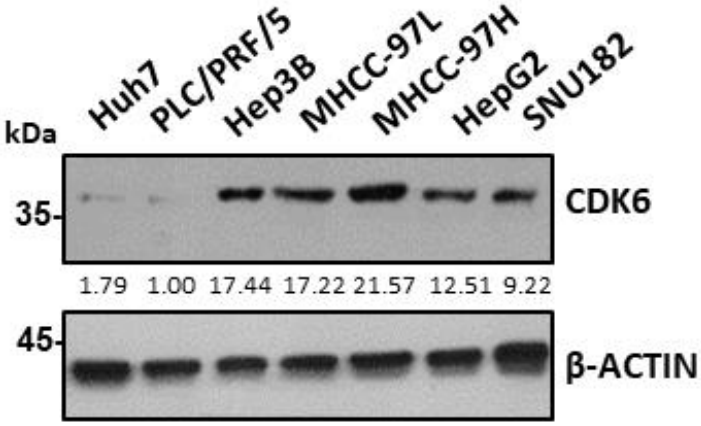

Supplementary Fig. 3. **Expression of CDK6 in a panel of HCC cell lines.** By western blot analysis, PLC/PRF/5 showed the lowest expression of CDK6 among the seven HCC cell lines. Expression of CDK6 was normalized to β-actin and expressed in fold change relative to PLC/PRF/5 (n=2 independent experiments). Source data are provided as a Source Data file.

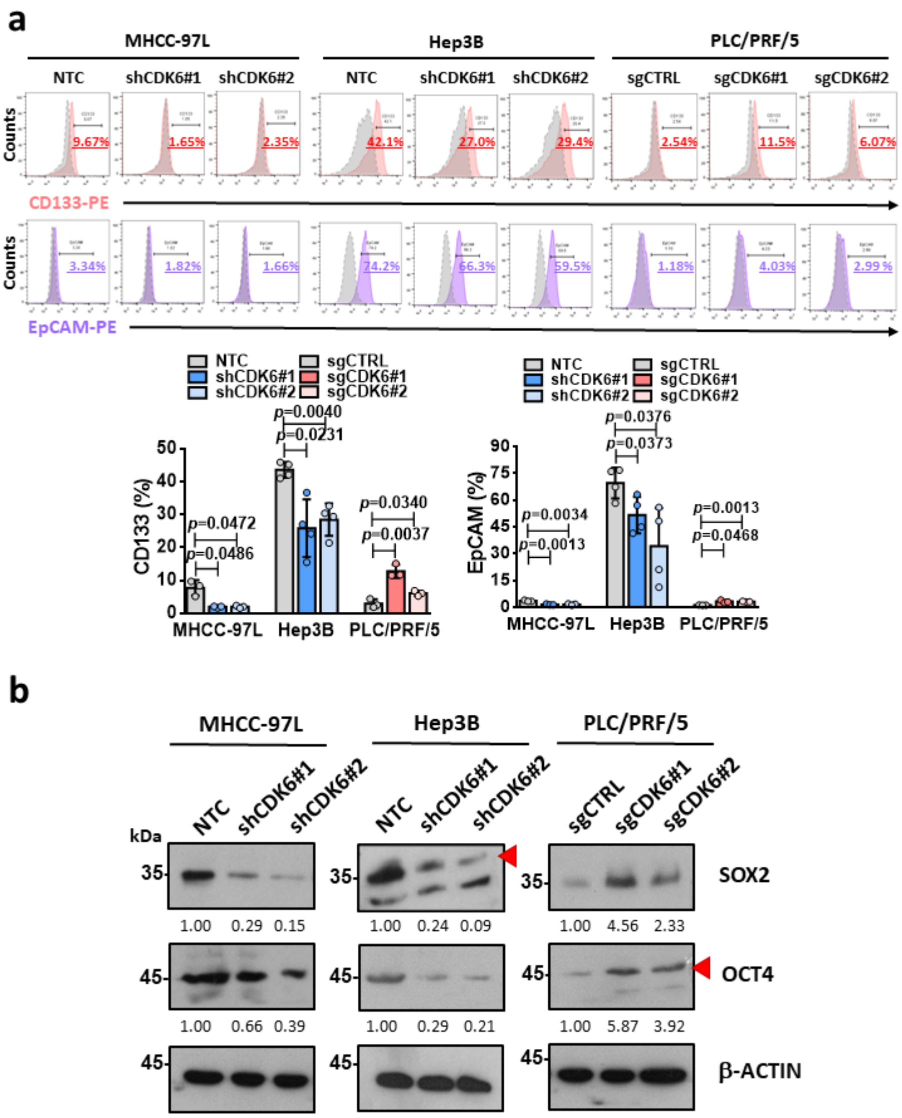

Supplementary Fig. 4. The role of CDK6 in the regulation of liver CSCs. a Alternations of CDK6 in HCC cell lines changed the expression of CD133 and EpCAM by flow cytometry analysis (MHCC-97L and PLC/PRF/5: *n*=3 and Hep3B: *n*=4 independent experiments; two-tailed *t* test). **b** By western blot analysis, the expression of pluripotent stem cell markers including SOX2 and OCT4 was suppressed upon CDK6 repression, while opposite effect was observed in two sgCDK6 clones. Expression of SOX2 and OCT4 was normalized to β-actin and expressed in fold change with respective to MHCC-97L NTC, Hep3B NTC and PLC/PRF/5 sgCTRL. Representative images from *n*=3 independent experiments. Data was presented as mean ± standard deviation. Source data are provided as a Source Data file.

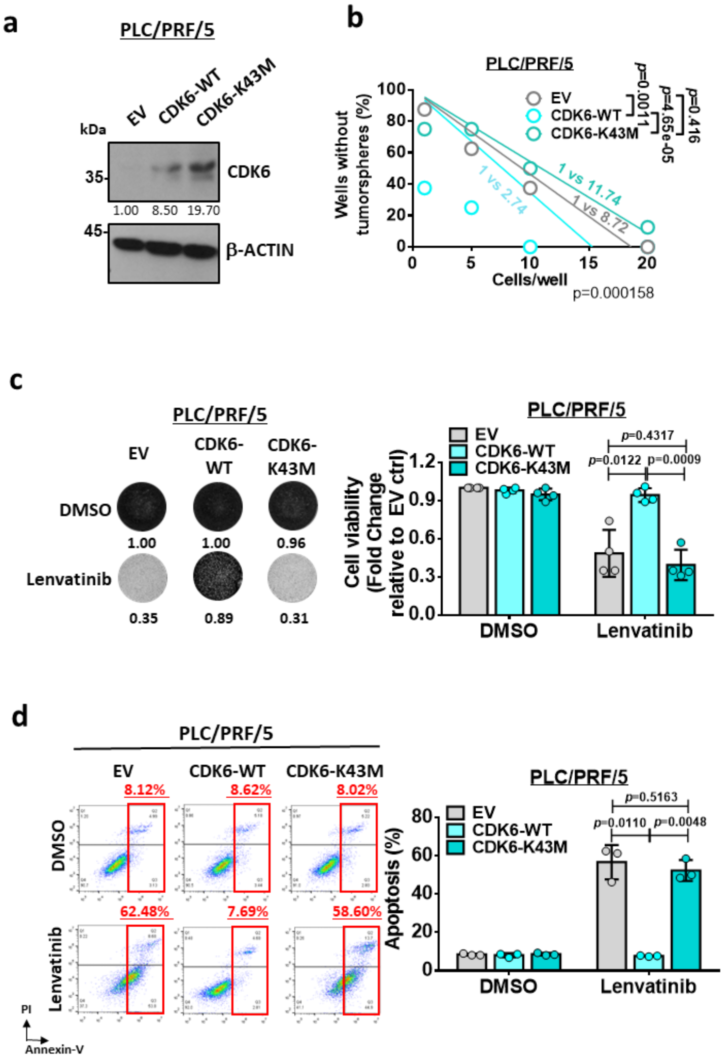

Supplementary Fig. 5. **The effect of CDK6 on cancer stemness and lenvatinib resistance is dependent on its kinase activity.** **a** The CDK6 protein levels in the empty vector (EV), wild-type CDK6 (CDK6-WT), CDK6 kinase dead-mutant (CDK6-K43M) PLC/PRF/5 cells. **b** *In vitro* limiting dilution assay showed enhanced stem cell frequency in CDK6-WT but not in CDK6-K43M (*n*= 2 independent experiments, one-sided extreme limiting dilution assay). **c** Long-term colony formation assay of EV, CDK6-WT and CDK6-K43M. Cells were grown in the presence of DMSO or lenvatinib at 40 µM, fixed and stained (*n*=4 independent experiments, two-tailed *t* test). **d** The apoptosis of EV, CDK6-WT and CDK6-K43M upon administration of lenvatinib at 40 µM (*n*=3 independent experiments, two-tailed *t* test). Data was presented as mean ± standard deviation. Source data are provided as a Source Data file.

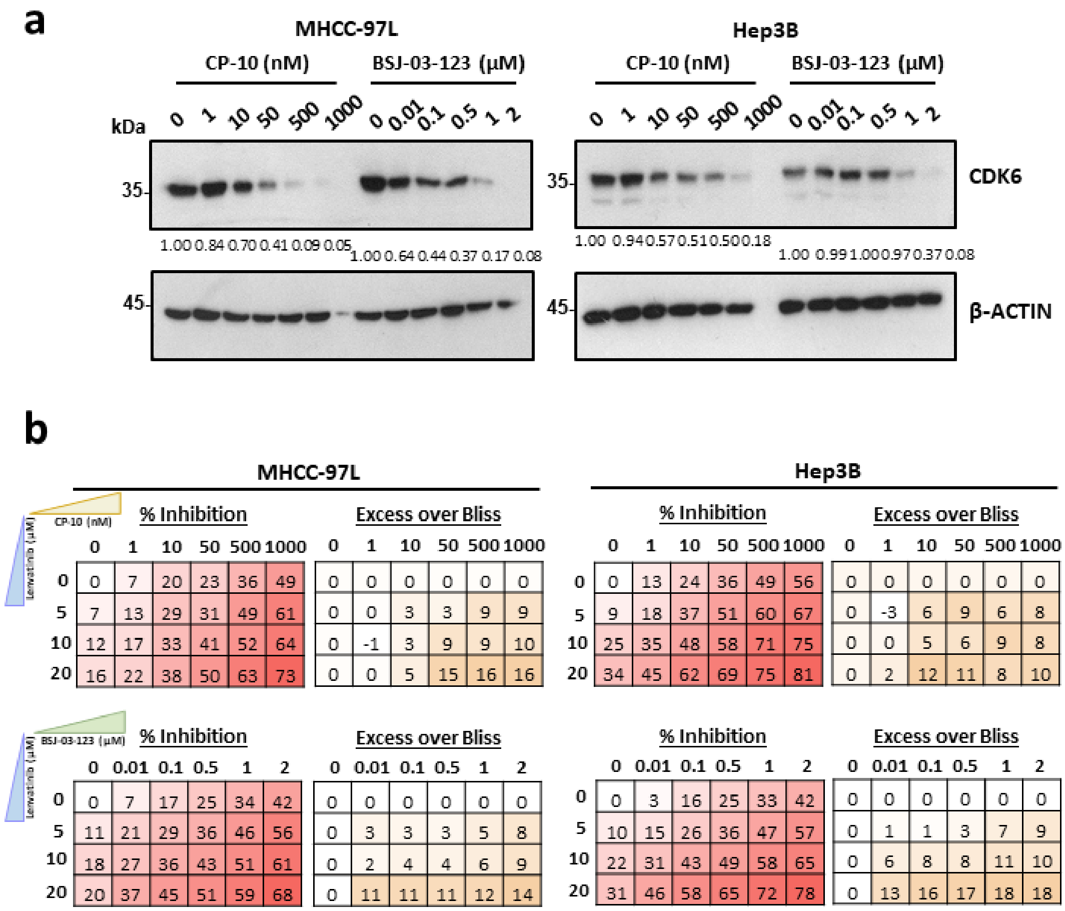

Supplementary Fig. 6. **The effect of CDK6 PROTAC degraders on high CDK6-expressing HCC cell lines. a** CP-10 at 500 nM while BSJ-03-123 at 1 μM effectively degraded the endogenous CDK6 in these two cell lines (*n*=2 independent experiments). **b** Combined treatment of lenvatinib with either CP-10 or BSJ-03-123 synergistically inhibited the growth of these two high CDK6-expressing HCC cell lines. Cells were treated with the indicated combination at different doses for 5 days. Cell viability was measured using MTT assay (*n*=3 independent experiments). Positive value in excess over Bliss indicated a synergistic effect in the combined treatment.

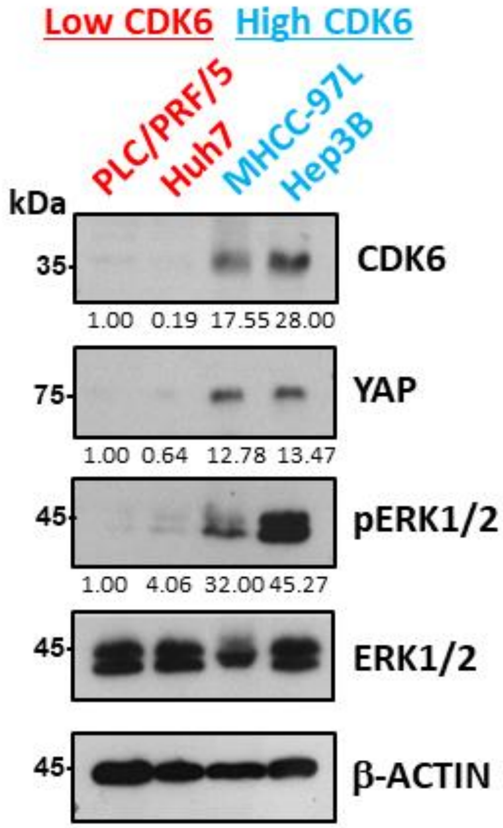

Supplementary Fig. 7. Upregulation of pERK1/2 and YAP in high CDK6-expressing HCC cells. By western blot analysis, expression of YAP and pERK1/2 was found to be more abundantly expressed in high CDK6-expressing HCC cells when compared with low counterparts. The expression of each target was normalized to β-actin and pERK1/2 was normalized to total ERK1/2. Expression indicated in form of fold changes relative to PLC/PRF/5 (*n*=2 independent experiments). Source data are provided as a Source Data file.

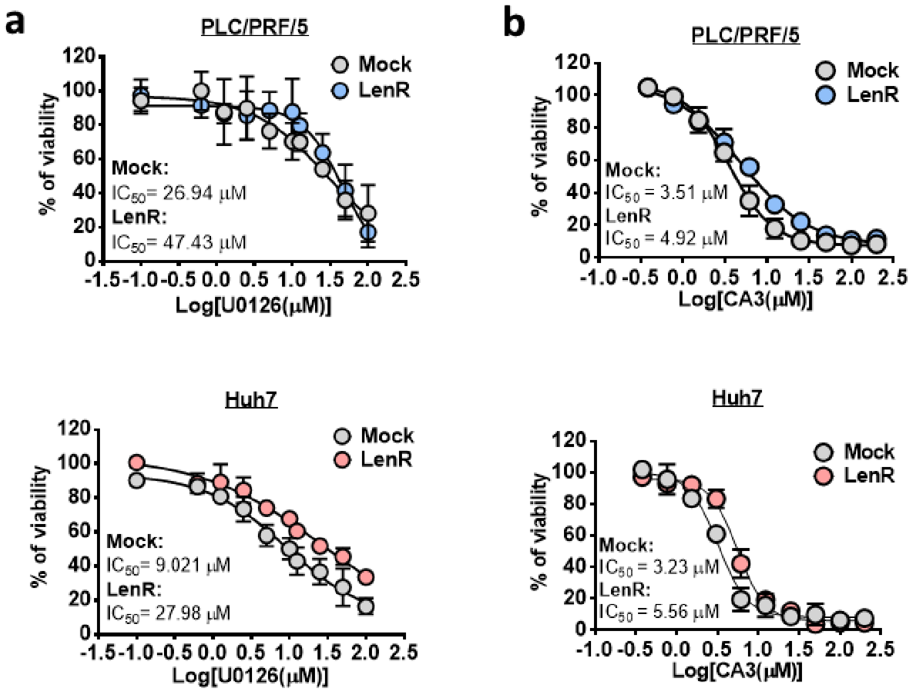

Supplementary Fig. 8. **The effect of U0126 and CA3 on mock and lenvatinib-resistant PLC/PRF/5 and Huh7 cells. a** IC_{50} of U0126 of mock and LenR PLC/PRF/5 and Huh7 was determined by MTT assay (*n*=3 independent experiments). **b** IC_{50} of CA3 of mock and LenR PLC/PRF/5 and Huh7 was determined by MTT assay (*n*=3 independent experiments). Data was presented as mean ± standard deviation. Source data are provided as a Source Data file.

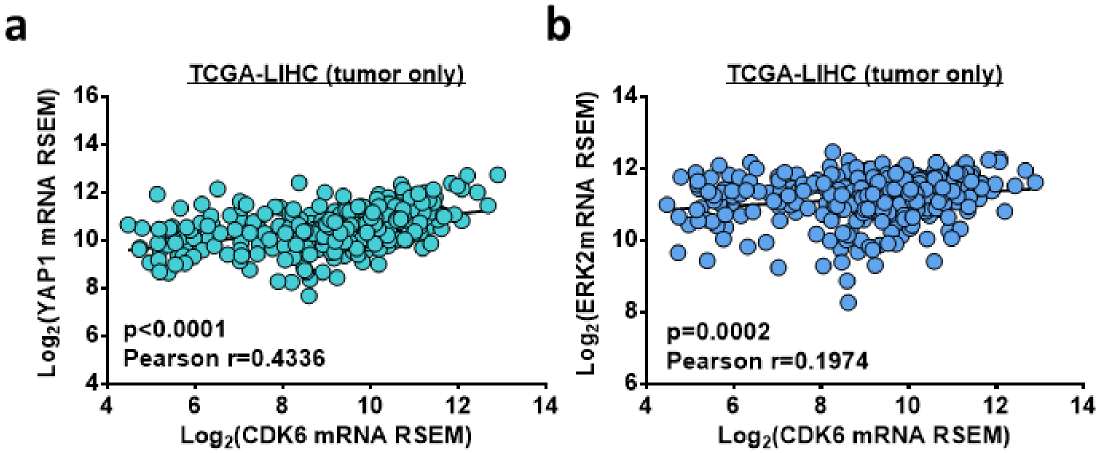

Supplementary Fig. 9. **The correlation of *CDK6* with *YAP1* and *ERK2* in a cohort of HCC patients.** **a** *CDK6* was positively correlated with *YAP1* in 371 HCC clinical samples (r=0.4336, p<0.0001). **b** *CDK6* was positively correlated with *ERK2* in 371 HCC clinical samples (r=0.1974, p=0.0002). TCGA data extracted from cBioportal (1-2). Source data are provided as a Source Data file.

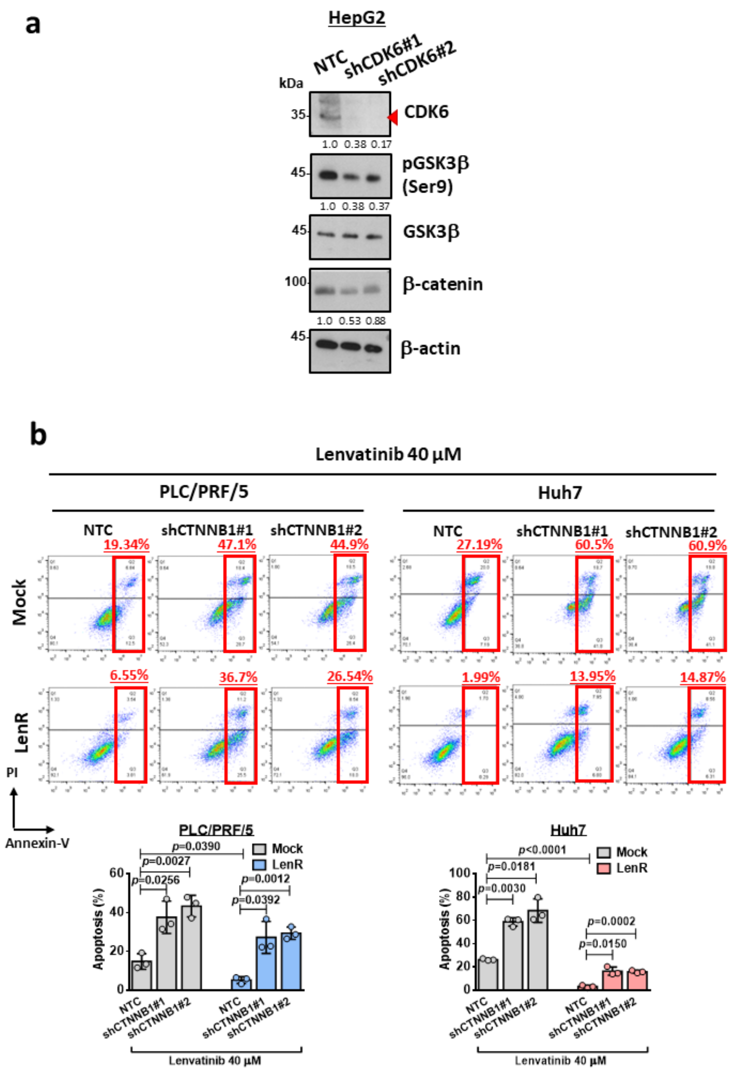

Supplementary Fig. 10. The role of CDK6 on Wnt/β-catenin signaling in HepG2 cells and effect of β-catenin suppression on lenvatinib resistance in mock and lenvatinib-resistant HCC cells. a Knockdown of CDK6 by lentiviral-based shRNA approach inhibited expression of phosphorylation of GSK3β at Serine 9 and β-catenin in HepG2 cells (*n*=2 independent experiments). **b** Knockdown of CTNNB1 by lentiviral-based shRNA approach LenR PLC/PRF/5 and Huh7 cells to lenvatinib treatment (*n*=3 independent experiment, two-tailed *t* test). The expression of each target was normalized to β-actin and pGSK3β was normalized to total GSK3β. Data was presented as mean ± standard deviation. Source data are provided as a Source Data file.

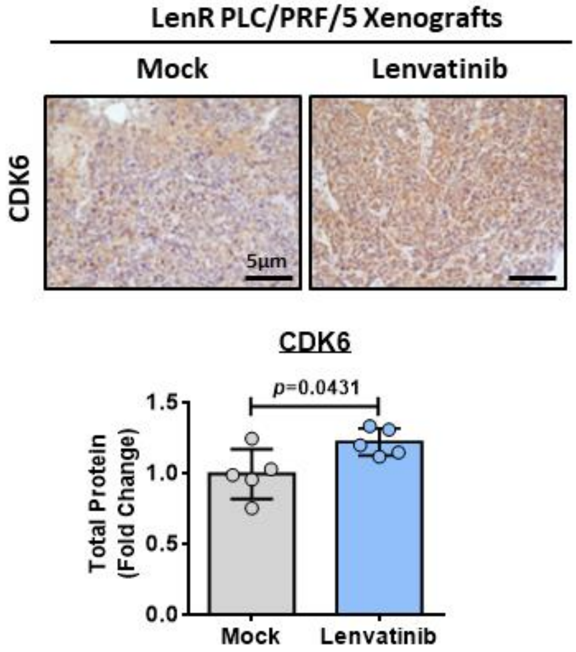

Supplementary Fig. 11. **Immunohistochemical staining of CDK6 in mock and LenR PLC/PRF/5 xenografts.** High CDK6 expression was observed in LenR PLC/PRF/5 xenograft tumors. Further increase in CDK6 protein expression was observed upon lenvatinib treatment at 30 mg/kg for 21 days (*n*=5, two-tailed *t* test). Scale bar=5 µm. Data was presented as mean ± standard deviation. Source data are provided as a Source Data file.

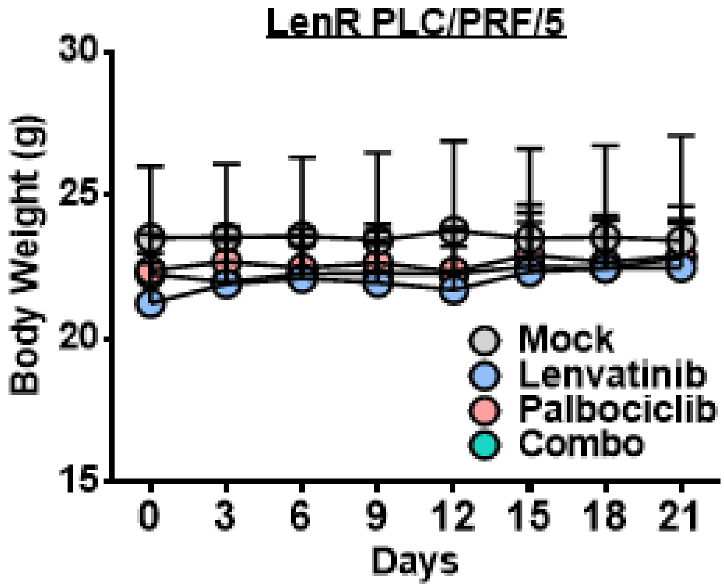

Supplementary Fig. 12. No notable change in body weight was observed after combined treatment for LenR PLC/PRF/5 xenograft of nude mice (*n*=5 mice for in each mock, lenvatinib and palbociclib group while *n*=6 mice for combo group). Source data are provided as a Source Data file.

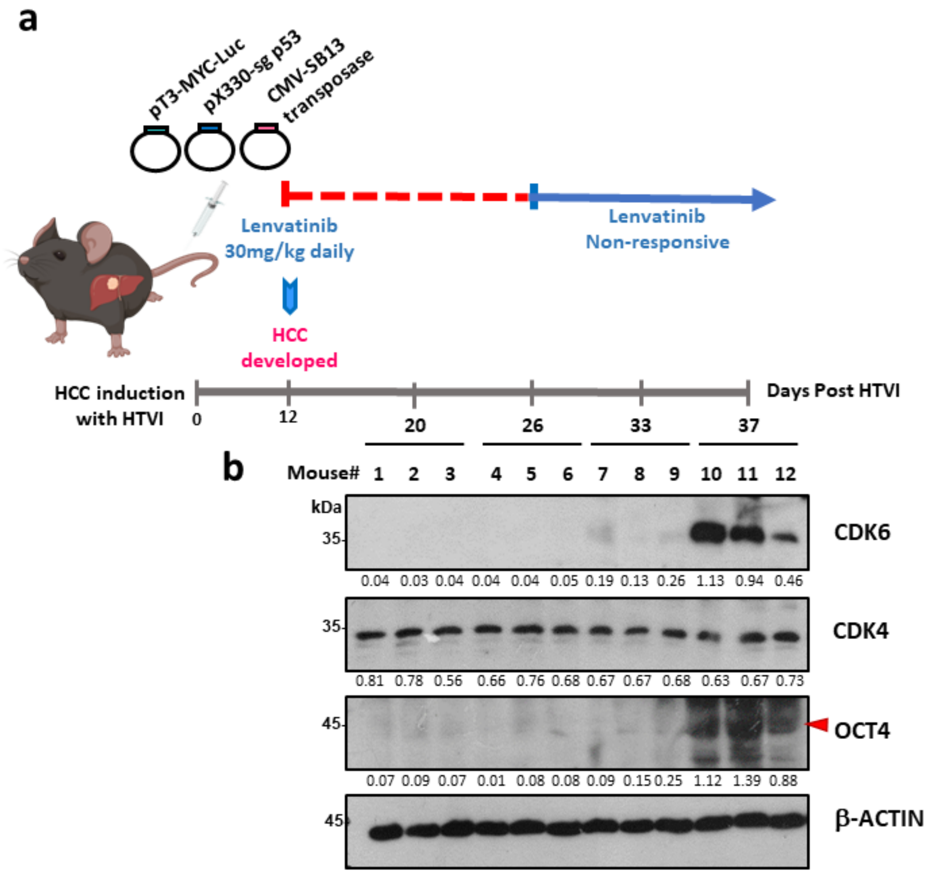

Supplementary Fig. 13. Protein expression of CDK6 and OCT4 was induced during the acquisition of lenvatinib resistance in *Trp53*^{KO}/*MYC*^{OE} HCC mouse model. a Mice were sacrificed at the indicated time points (Day 20, Day 26, Day 33 and Day 37) upon HTVI. The tumor lysate was subjected to western blot analysis. **b** Expression of CDK6 and OCT4 was upregulated starting on Day 33 upon HTVI, while CDK4 expression showed no significant alteration during the same period of time. Intensity of each target was quantified and normalized to β-actin expression. Source data are provided as a Source Data file.

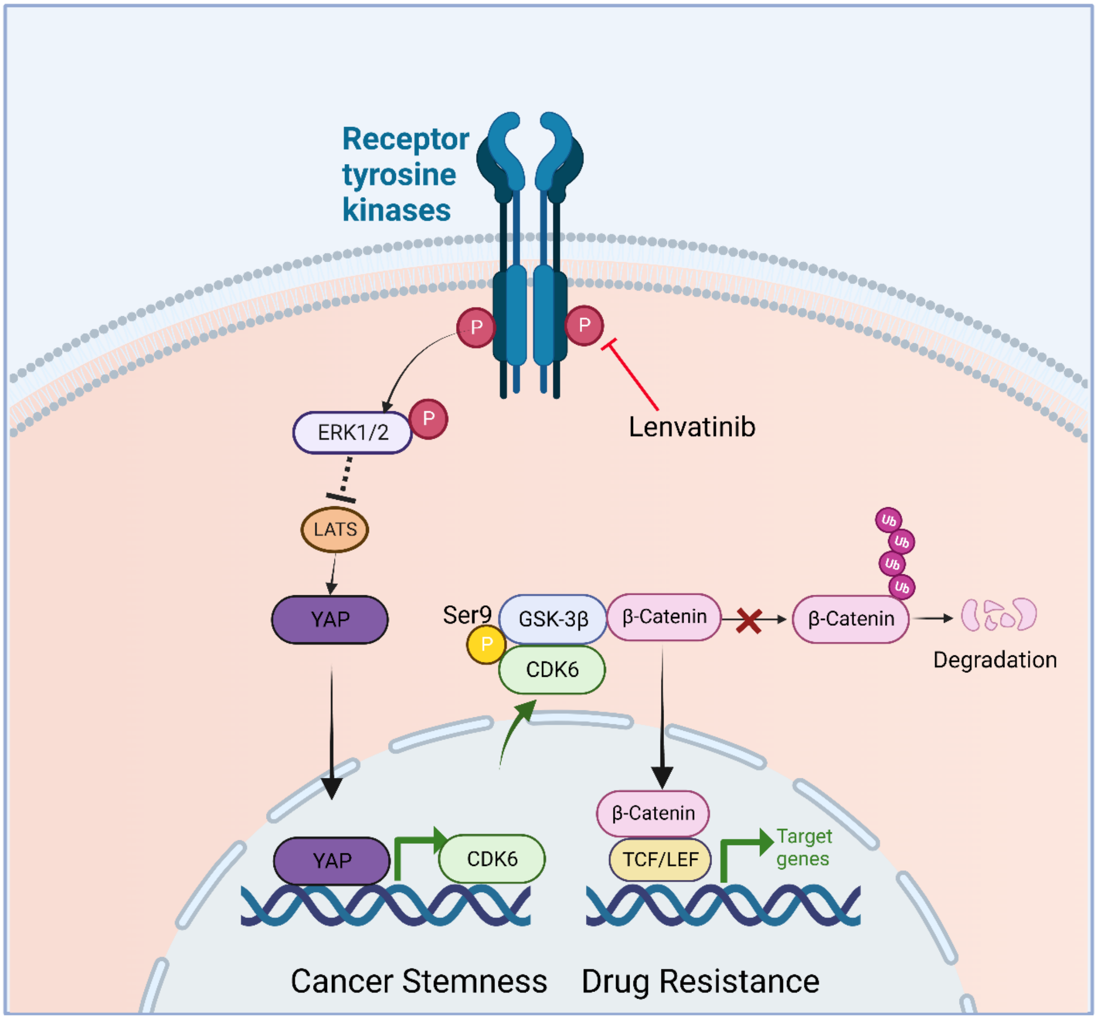

Supplementary Fig. 14. Schematic diagram showing the molecular mechanism for the development of lenvatinib resistance in HCC cells.

Supplementary Tables

Supplementary Table 1. Clinicopathological features of the lenvatinib-sensitive and resistant HCC patients.

	**Lenvatinib response**	**HBV status**	**Tumor Size**	**No. of nodules**	**No. of lymph node metastasis**	**Presence of venous infiltration**	**Presence of extrahepatic metastasis**
**1**	Resistant	+ve	84×71mm	1	2	Yes	No
**2**	Resistant	neg	60×56mm	3+	0	No	No
**3**	Resistant	+ve	BCLC Stage A	1	1	No	No
4	Sensitive	+ve	68×59mm	2	0	No	No
5	Sensitive	+ve	116×121mm	3+	0	No	No
6	Sensitive	+ve	118×76mm	1	0	No	No
7	Sensitive	+ve	148×112mm	1	0	No	No

Supplementary Table 2. Effect of CDK6 suppression on tumorigenicity of MHCC-97L and Hep3B cells using lentiviral based knockdown approach, PLC/PRF/5 cells with CDK6 overexpression. **a** Subcutaneous *in vivo* tumor development in NOD/SCID mice of shCDK6 cells and non-target control (NTC) cells from MHCC-97L. **b** Subcutaneous *in vivo* tumor development in NOD/SCID mice of shCDK6 cells and NTC cells from Hep3B. **c** Subcutaneous *in vivo* tumor development in NOD/SCID mice of sgCDK6 cells and sgControl (sgCTRL) cells from PLC/PRF/5. Significance were calculated by one-sided extreme limiting dilution analysis.

a Primary engraftment of MHCC-97L cells

	**Tumor incidence rate**			**Extreme limiting dilution**		
	$1X10^3$ cells	$5x10^3$ cells	$1x10^4$ cells	Estimated CSC frequency	95% CI	*p*-value
**NTC**	3/5	5/5	5/5	1/1023	1/2789-1/375	
**shCDK6#1**	1/5	2/5	2/5	1/12571	1/31334-1/5043	6.68e-05
**shCDK6#2**	1/5	2/5	3/5	1/9407	1/21699-1/4079	0.000355

b Primary engraftment of Hep3B cells

	**Tumor incidence rate**			**Extreme limiting dilution**		
	$0.5X10^6$ cells	$1x10^6$ cells	$1.5x10^6$ cells	Estimated CSC frequency	95% CI	*p*-value
**NTC**	4/6	5/6	6/6	1/458112	1/857498-1/244743	
**shCDK6#1**	1/6	3/6	3/6	1/1939766	1/4114336-1/914532	0.00256
**shCDK6#2**	0/6	3/6	3/6	1/2317862	1/5162383-1/1040699	0.000992

c Primary engraftment of PLC/PRF/5 cells

	**Tumor incidence rate**			**Extreme limiting dilution**		
	$1X10^3$ cells	$5x10^3$ cells	$1x10^4$ cells	Estimated CSC frequency	95% CI	*p*-value
**sgCTRL**	1/5	1/5	1/5	1/23854	1/76406-1/7448	
**sgCDK6#1**	2/5	3/5	3/5	1/6476	1/13960-1/3004	0.0426
**sgCDK6#2**	3/5	5/5	5/5	1/1023	1/2789-1/375	2.44e-06

Supplementary Table 3. The clinicopathological features of 51 HCC patients.

Clinical Features	Total no. of cases
**Gender**	
Male	47
Female	4
**Age (Median=52)**	
≤52	27
≥52	24
**No. of nodules**	
Single	33
Multiple	18
**Tumor size**	
<5 cm	14
≥5 cm	37
**Encapsulation**	
No	33
Yes	18
**HBsAg**	
No	11
Yes	40
**AFP elevation**	
<20 ng/ml	12
≥20 ng/ml	39
**TNM**	
Stage I/II	31
Stage III/IV	20
**Differentiation**	
Well/Moderate	34
Poor	17
**Cirrhosis**	
No	2
Yes	49
**Venous infiltration**	
**No**	43
**Yes**	8
**Relapse**	
No	25
Yes	26

Supplementary Table 4. shRNA and sgRNA sequences.

	Sequence (5'-3')
**NTC**	CCGGTTGTGCTCTTCATCTTGTTGCCGGCAACAAGATGAAGAGCACCAATTTTTG
**shCDK6#1**	CGATCAAGACTTGACCACTTA
**shCDK6#2**	GACCTGGAAAGGTGCAAAGAA
**sgCTRL**	GATACGTCGGTACCGGACCG
**sgCDK6#1**	GAGCCCCCACCCACATTCTG
**sgCDK6#2**	GCATCGGGTTACAGGGTCCT
**shCTNNB1#1**	GCGCATGGAAGAAATAGTTGAA
**shCTNNB1#2**	GCCTTTAGCTGTATTGTCTGAA

Supplementary Table 5. Primer sequences for qRT-PCR.

Genes	Forward primer (5'-3')	Reverse primer (5'-3')
**CDK6**	TCTTCATTCACACCGAGTAGTGC	TGAGGTTAGAGCCATCTGGAAA
**AXIN2**	CAGCGAGTATTACTGCTACTCGAAA	TTTTTTGTGCTTTGGGCACTATG
**CMYC**	CGTCCTCGGATTCTCTGCTC	GCTGGTGCATTTTCGGTTGT
**CCND1**	AGCTCCTGTGCTGCGAAGTGGAAAC	AGTGTTCAATGAAATCGTGCGGGGT
**HPRT**	CCTGGCGTCGTGATTAGTGAT	AGACGTTCAGTCCTGTCCATAA

References

1. Gao, J., *et al*. Integrative analysis of complex cancer genomics and clinical profiles using the cBioPortal. *Sci Signal* **6**,pl1 (2013).
2. Cerami, E., *et al*. The cBio cancer genomics portal: an open platform for exploring multidimensional cancer genomics data. *Cancer Discov* **2**,401-404 (2012).
