# Supplementary material for: Broad-spectrum kinome profiling identifies CDK6 upregulation as a driver of lenvatinib resistance in hepatocellular carcinoma
Source: Nat Commun. 2023 Oct 23;14:6699. doi: 10.1038/s41467-023-42360-w (PMC10593849; doi:10.1038/s41467-023-42360-w)
Supplement: Supplementary file 2 — Reporting Summary [file 41467_2023_42360_MOESM2_ESM.pdf]

nature portfolio

Corresponding author(s): Prof. Terence KW LEE & Dr. Q Zhao

Last updated by author(s): Oct 9, 2023

Reporting Summary

Nature Portfolio wishes to improve the reproducibility of the work that we publish. This form provides structure for consistency and transparency in reporting. For further information on Nature Portfolio policies, see our Editorial Policies and the Editorial Policy Checklist.

Statistics

For all statistical analyses, confirm that the following items are present in the figure legend, table legend, main text, or Methods section.

n/a	Confirmed	
☐	☒	The exact sample size (*n*) for each experimental group/condition, given as a discrete number and unit of measurement
☒	☐	A statement on whether measurements were taken from distinct samples or whether the same sample was measured repeatedly
☐	☒	The statistical test(s) used AND whether they are one- or two-sided<br>*Only common tests should be described solely by name; describe more complex techniques in the Methods section.*
☒	☐	A description of all covariates tested
☐	☒	A description of any assumptions or corrections, such as tests of normality and adjustment for multiple comparisons
☐	☒	A full description of the statistical parameters including central tendency (e.g. means) or other basic estimates (e.g. regression coefficient) AND variation (e.g. standard deviation) or associated estimates of uncertainty (e.g. confidence intervals)
☐	☒	For null hypothesis testing, the test statistic (e.g. *F*, *t*, *r*) with confidence intervals, effect sizes, degrees of freedom and *P* value noted<br>*Give P values as exact values whenever suitable.*
☒	☐	For Bayesian analysis, information on the choice of priors and Markov chain Monte Carlo settings
☒	☐	For hierarchical and complex designs, identification of the appropriate level for tests and full reporting of outcomes
☒	☐	Estimates of effect sizes (e.g. Cohen's *d*, Pearson's *r*), indicating how they were calculated

Our web collection on statistics for biologists contains articles on many of the points above.

Software and code

Policy information about availability of computer code

Data collection	Accuri C6 flow cytometer and FACSDiva software (BD Biosciences) was used to collect flow cytometry data.<br>QuantStudio 7 Flex Real Time PCR System (Applied Biosystems) was used to collect qPCR data.<br>Exploris 480 Orbitrap mass spectrometer (Thermo Fisher Scientific) coupled with an UltiMate 3000 UPLC system (Thermo Fisher Scientific) were used for LC-MS/MS analyses.<br>TCS SPE (Leica) confocal microscope was used to collect multiple immunofluorescence images.<br>FACSAriaIII cell sorter (BD Biosciences) was using for sorting of CD45+ cells for scRNA sequencing. Libraries were prepared and sequenced on a NovaSeq platform (Illumina).
Data analysis	FlowJo (Version 10) was used for analysis of flow cytometry data.<br>QuantStudio Real-Time PCR System software (version 1.3) was used for analysis of qPCR data.<br>GraphPad Prism (Version 6) was used for analysis of data and carry out statistical analysis.<br>Tumor-initiating cell frequency was calculated using Extreme Limiting Dilution Analysis (ELDA) software.<br>Protein-protein interaction analysis from proteomics data was performed using STRING platform (version 11.0b).<br>The TMT data from phosphoproteome analysis were searched against the Homo sapiens UniProt database (version June 2020, 20368 entries) using the SEEQIUEST algorithm (Proteome Discoverer 2.4, Thermo Fisher Scientific).<br>The acquired label-free DDA data were searched against the Homo sapiens UniProt database (version June 2020) using the SEQUEST algorithm (Proteome Discoverer 2.4, Thermo Fisher Scientific).<br>GSEA (version 4.0.3) with hallmark gene sets database was used for GSEA experiments.<br>ImageJ (version 1.51k) was used to analyze immunohistochemistry and multiple immunofluorescence images.<br>Cell Ranger Single Cell software (version 5.0.1), Seurat R package (version 4.1.1), SingleR package (version 2.0.0) and TILPRED (version 1.0)

were used for analysis of scRNA sequencing data.

For manuscripts utilizing custom algorithms or software that are central to the research but not yet described in published literature, software must be made available to editors and reviewers. We strongly encourage code deposition in a community repository (e.g. GitHub). See the Nature Portfolio guidelines for submitting code & software for further information.

Data

Policy information about availability of data

All manuscripts must include a data availability statement. This statement should provide the following information, where applicable:

- Accession codes, unique identifiers, or web links for publicly available datasets
- A description of any restrictions on data availability
- For clinical datasets or third party data, please ensure that the statement adheres to our policy

The mass spectrometry proteomics data have been deposited to the ProteomeXchange Consortium via the PRIDE partner repository with the dataset identifier PXD038152. scRNA sequencing data is available at GSE218010. Transcriptome profiling data of human normal and cirrhotic livers, early to advanced stage of HCC dataset (GSE25097) was directly access through GEO website (NCBI).

Human research participants

Policy information about studies involving human research participants and Sex and Gender in Research.

Reporting on sex and gender

1. Levatinib-sensitive and lenvatinib-resistant HCC tumor samples
All subjects' sex were consistent with their gender. Participants were Chinese male, aged from 43-57.

2. Tissue microarray of HCC samples
All subjects' sex were consistent with their gender. All participants were Chinese. Among the 51 samples collected, 47 participants were male while 4 were female. Median age of the participants was 52.

Population characteristics

1. Levatinib-sensitive and lenvatinib-resistant HCC tumor samples
Participants were Chinese male, aged from 43-57.

2.2. Tissue microarray of HCC samples
Participants were Chinese of median age was 52.

Recruitment

1. Levatinib-sensitive and lenvatinib-resistant HCC tumor samples
Primary HCC patients were recruited by Tongji Hospital, Tongji Medical College, Huazhong Univerisity of Science and Technology. There is no potential bias for recruiting patient cohort. Seven archived paraffin-embedded pathological specimens from primary HCC patients were collected along with complete clinical and pathological data at the Tongji Hospital, Tongji Medical College, Huazhong University of Science and Technology, Wuhan, China. All samples were anonymous.

2. Tissue microarray of HCC samples
Primary HCC patients were recuited by Sun Yat-sen University Cancer Center in Gua.ngzhou, China. There is no potential bias for recruiting patient cohort.51 archived paraffin-embedded pathological specimens from primary HCC patients were collected along with complete clinical and pathological data at the Sun Yat-sen University Cancer Center. All samples were anonymous.

Ethics oversight

1. Levatinib-sensitive and lenvatinib-resistant HCC tumor samples
This study was approved by the Institutional Review Board for ethical review from Huazhong University of Science and Technology, Wuhan, China (2019s1096). The procurement of all clinical information has received consent from patients.

2. Tissue microarray of HCC samples
Institutional Review Board and Human Ethics Committee of Sun Yat-sen University Cancer Center approved collection and usage of tissue microarray. All patients participated with informed consent. This study was approved by the Institute Research Medical Ethics Committee (G-2022-105-01).

Note that full information on the approval of the study protocol must also be provided in the manuscript.

Field-specific reporting

Please select the one below that is the best fit for your research. If you are not sure, read the appropriate sections before making your selection.

☒ Life sciences ☐ Behavioural & social sciences ☐ Ecological, evolutionary & environmental sciences

For a reference copy of the document with all sections, see nature.com/documents/nr-reporting-summary-flat.pdf

Life sciences study design

All studies must disclose on these points even when the disclosure is negative.

Sample size	We did not perform sample size calculations and sample sizes were chosen according to accepted standards in the field of study (Naegle K, Gough NR, Yaffe MB. Criteria for biological reproducibility: what does "n" mean? Sci Signal. 2015 Apr 7;8(371):fs7. doi: 10.1126/scisignal.aab1125.). Individual data points from biological replicates were shown in each figure. For animal studies, preliminary experiments were performed to determine the variation in xenograft (subcutaneous inoculation) or liver tumor (hydrodynamic tail vein injection) growth rate and response to treatment. The scRNA sequencing was performed from lenvatinib resistant mouse treated either with lenvatinib or combined lenvatinib and palbociclib, with one mouse included for each set of data.
Data exclusions	No data was excluded from this study.
Replication	All experiments were repeated three times or more, with the exception of the following:<br>1. XO44 kinase pull down mass spectrometry analysis (n=1)<br>2. parallel reaction monitoring (PRM) (n=2)<br>The number of biological replicates and independent experiments are stated in the figure legend. All attempts at the replication were successful.
Randomization	All mice were randomly assigned to treatment groups to ensure similar initial size of xenografts (subcutaneous inoculation) or similar initial bioluminescent signal intensity of liver tumors (hydrodynamic tail vein injection) before administration of treatment between groups.
Blinding	All authors were not blinded to group allocation, data collection or data analysis because the investigators were responsible for performing the experiment, collecting and labelling the samples, and analysis of data. For analysis of sequencing data, blinding was not necessary.We have planned the analysis pipeline before the acquisition of sequencing data.

Reporting for specific materials, systems and methods

We require information from authors about some types of materials, experimental systems and methods used in many studies. Here, indicate whether each material, system or method listed is relevant to your study. If you are not sure if a list item applies to your research, read the appropriate section before selecting a response.

Materials & experimental systems

n/a	Involved in the study
☐	☒ Antibodies
☐	☒ Eukaryotic cell lines
☒	☐ Palaeontology and archaeology
☐	☒ Animals and other organisms
☐	☒ Clinical data
☒	☐ Dual use research of concern

Methods

n/a	Involved in the study
☒	☐ ChIP-seq
☐	☒ Flow cytometry
☒	☐ MRI-based neuroimaging

Antibodies

Antibodies used	β-actin (AC-74) (#A5316, Sigma Aldrich)<br>β-catenin (D10A8) (#8480S, Cell Signaling Technology)<br>CD4 (D7D2Z) (#25229S, Cell Signaling Technology)<br>CD8α (D4W2Z) (#98941S, Cell Signaling Technology)<br>CDK4 (D9G3E) (#12790, Cell Signaling Technology)<br>CDK4 [EPR2513Y] (ab68266, Abcam)<br>CDK6 (D4S8S) (#13331, Cell Signaling Technology)<br>CDK6 [EPR4515] (ab124821, Abcam)<br>CDK6 (ab151247, Abcam)<br>FOXP3 (5H10L18) (#700914, Thermo Scientific)<br>GSK3β (H-76) (sc9166, Santa Cruz)<br>GSK3β (clone 7) (#610202, clone 7, BD Transduction Laboratories)<br>p-GSK3β (Ser9) (D3A4) (#9322S, Cell Signaling Technology)<br>HA (1F5C6) (#66061-1-Ig-AP, clone 1C1D2, Proteintech)<br>MAP3K7 [EPR5984] (ab109526, Abcam)<br>p44/42 MAPK (#9102S, Cell Signaling Technology)<br>p-p44/42 MAPK (#9101S, Cell Signaling Technology)<br>OCT4 (C-10) (sc5279, Clone C-10, Santa Cruz)<br>PCNA [PC10] (ab29, Abcam)<br>PD-1 (D7D5W) (#84651S, Cell Signaling Technology)

RPS6KA4 (D41A4) (#3679, Cell Signaling Technology)
SOX2 (D6D9) (#3579, Cell Signaling Technology)
YAP (D8H1X) (#14074S Cell Signaling Technology)
p-YAP(Ser127) (D9W2I) (#13008, Cell Signaling Technology)

Validation

All antibodies are validated by the manufacturer as suitable for the applications. The product size in Western Blot experiments were confirmed by comparison to protein size ladder.

β-actin (AC-74) (#A5316, Sigma Aldrich): https://www.sigmaaldrich.com/HK/en/product/sigma/a5316

β-catenin (D10A8) (#8480S, Cell Signaling Technology): https://www.cellsignal.com/products/primary-antibodies/b-catenin-d10a8-xp-rabbit-mab/8480

CD4 (D7D2Z) (#25229S, Cell Signaling Technology): https://www.cellsignal.com/products/primary-antibodies/cd4-d7d2z-rabbit-mab/25229

CD8α (D4W2Z) (#98941S, Cell Signaling Technology): https://www.cellsignal.com/products/primary-antibodies/cd8a-d4w2z-xp-rabbit-mab/98941

CDK4 (D9G3E) (#12790, Cell Signaling Technology): https://www.cellsignal.com/products/primary-antibodies/cdk4-d9g3e-rabbit-mab/12790

CDK4 [EPR2513Y] (ab68266, Abcam): https://www.abcam.com/products/primary-antibodies/cdk4-antibody-epr2513y-ab68266.html

CDK6 (D4S8S) (#13331, Cell Signaling Technology): https://www.cellsignal.com/products/primary-antibodies/cdk6-d4s8s-rabbit-mab/13331

CDK6 [EPR4515] (ab124821, Abcam): https://www.abcam.com/products/primary-antibodies/cdk6-antibody-epr4515-ab124821.html

CDK6 (ab151247, Abcam): https://www.abcam.com/products/primary-antibodies/cdk6-antibody-ab151247.html

FOXP3 (5H10L18) (#700914, Thermo Scientific): https://www.thermofisher.com/antibody/product/FOXP3-Antibody-clone-5H10L18-Recombinant-Monoclonal/700914

GSK3β (H-76) (sc9166, Santa Cruz): https://datasheets.scbt.com/sc-9166.pdf

GSK3β (clone 7) (#610202, clone 7, BD Transduction Laboratories): https://www.bdbiosciences.com/en-nz/products/reagents/microscopy-imaging-reagents/immunofluorescence-reagents/purified-mouse-anti-gsk-3.610202

p-GSK3β (Ser9) (D3A4) (#9322S, Cell Signaling Technology): https://www.cellsignal.com/products/primary-antibodies/phospho-gsk-3-beta-ser9-d3a4-rabbit-mab/9322?_requestid=4256347

HA (1F5C6) (#66061-1-Ig-AP, clone 1C1D2, Proteintech): https://www.ptglab.com/products/HA-Tag-Antibody-66006-1-Ig.htm

MAP3K7 [EPR5984] (ab109526, Abcam): https://www.abcam.com/products/primary-antibodies/tak1-antibody-epr5984-ab109526.html

p44/42 MAPK (#9102S, Cell Signaling Technology): https://www.cellsignal.com/products/primary-antibodies/p44-42-mapk-erk1-2-antibody/9102

p-p44/42 MAPK (#9101S, Cell Signaling Technology): https://www.cellsignal.com/products/primary-antibodies/phospho-p44-42-mapk-erk1-2-thr202-tyr204-antibody/9101

OCT4 (C-10) (sc5279, Clone C-10, Santa Cruz): https://www.scbt.com/p/oct-3-4-antibody-c-10

PCNA [PC10] (ab29, Abcam): https://www.abcam.com/products/primary-antibodies/pcna-antibody-pc10-ab29.html

PD-1 (D7D5W) (#84651S, Cell Signaling Technology): https://www.cellsignal.com/products/primary-antibodies/pd-1-intracellular-domain-d7d5w-xp-rabbit-mab/84651

RPS6KA4 (D41A4) (#3679, Cell Signaling Technology): https://www.cellsignal.com/products/primary-antibodies/msk2-d41a4-xp-rabbit-mab/3679

SOX2 (D6D9) (#3579, Cell Signaling Technology): https://www.cellsignal.com/product/productDetail.jsp?productId=3579

YAP (D8H1X) (#14074S Cell Signaling Technology): https://www.cellsignal.com/product/productDetail.jsp?productId=14074

p-YAP(Ser127) (D9W2I) (#13008, Cell Signaling Technology): https://www.cellsignal.com/products/primary-antibodies/phospho-yap-ser127-d9w2i-rabbit-mab/13008

Eukaryotic cell lines

Policy information about cell lines and Sex and Gender in Research

Cell line source(s)	MHCC-97L and MHCC-97H human HCC cell lines (Liver Cancer Institute, Fudan University); Hep3B, HepG2 and SNU-182 human HCC cell lines (America Type Culture Collection, Manassas, VA, USA); Huh7 and PLC/PRF/5 human HCC cell lines (Japan Cancer Research Bank); and 293FT (Invitrogen, Thermo Fisher Scientific, Waltham, USA).
Authentication	All cell lines used in this study were obtained between 2013 and 2016, and they were authenticated by morphological observation and STR DNA analysis with PowerPlex® 16HS kit (Promega) as well as tested for the absence of mycoplasma contamination (MycoAlert, Lonza)
Mycoplasma contamination	All cell lines were negatively for mycoplasma contamination and routinely tested for microplasma using PCR assay.
Commonly misidentified lines (See ICLAC register)	No commonly misidentified cell lines were used.

Animals and other research organisms

Policy information about studies involving animals; ARRIVE guidelines recommended for reporting animal research, and Sex and Gender in Research

Laboratory animals	Mouse (Mus musculus), BALB/cAnN-nu (Nude), 4 to 6 weeks<br>Mouse (Mus musculus), NOD/SCID, 4 to 6 weeks<br>Mouse (Mus musculus),wild-type C57BL/6J mice, 6 to 8 weeks
Wild animals	No wild animals were used in the study.
Reporting on sex	Male Mouse (Mus musculus), BALB/cAnN-nu (Nude), 4 to 6 weeks<br>Male Mouse (Mus musculus), NOD/SCID, 4 to 6 weeks<br>Male Mouse (Mus musculus),wild-type C57BL/6J mice, 6 to 8 weeks
Field-collected samples	No field collected samples were used in the study.
Ethics oversight	License to conduct experiments on animals was obtained from Department of Health, Hong Kong SAR.<br>The study protocol was approved by and performed in accordance with the Animal Subjects Ethics Sub-Committee (ASESC) at the Hong Kong Polytechnic University (Hong Kong, P.R. China).

Note that full information on the approval of the study protocol must also be provided in the manuscript.

Clinical data

Policy information about clinical studies
All manuscripts should comply with the ICMJEguidelines for publication of clinical research and a completedCONSORT checklist must be included with all submissions.

Clinical trial registration	*Provide the trial registration number from ClinicalTrials.gov or an equivalent agency.*
Study protocol	*Note where the full trial protocol can be accessed OR if not available, explain why.*
Data collection	*Describe the settings and locales of data collection, noting the time periods of recruitment and data collection.*
Outcomes	*Describe how you pre-defined primary and secondary outcome measures and how you assessed these measures.*

Flow Cytometry

Plots

Confirm that:

- [x] The axis labels state the marker and fluorochrome used (e.g. CD4-FITC).
- [x] The axis scales are clearly visible. Include numbers along axes only for bottom left plot of group (a 'group' is an analysis of identical markers).
- [x] All plots are contour plots with outliers or pseudocolor plots.
- [x] A numerical value for number of cells or percentage (with statistics) is provided.

Methodology

Sample preparation	HCC cells were stained with specific antibody in FACS buffers.
Instrument	BD Accuri C6 flow cytometer, FACSDiva software (BD Biosciences)
Software	FlowJo (Version 10)
Cell population abundance	The final target population consists of at least 10,000 cells
Gating strategy	The total population was visualized using FSC/SSC graph, and the main population was gated.<br>Subsequently, singlets were gated using FSH-H/FSC-A graph.<br>The singlets were then used for analysis.

☐ Tick this box to confirm that a figure exemplifying the gating strategy is provided in the Supplementary Information.
